# NOTUM inhibition increases endocortical bone formation and bone strength

**DOI:** 10.1038/s41413-018-0038-3

**Published:** 2019-01-08

**Authors:** Robert Brommage, Jeff Liu, Peter Vogel, Faika Mseeh, Andrea Y. Thompson, David G. Potter, Melanie K. Shadoan, Gwenn M. Hansen, Sabrina Jeter-Jones, Jie Cui, Dawn Bright, Jennifer P. Bardenhagen, Deon D. Doree, Sofia Movérare-Skrtic, Karin H. Nilsson, Petra Henning, Ulf H. Lerner, Claes Ohlsson, Arthur T. Sands, James E. Tarver, David R. Powell, Brian Zambrowicz, Qingyun Liu

**Affiliations:** 1 Lexicon Pharmaceuticals, The Woodlands, TX USA; 2 Centre for Bone and Arthritis Research, Institute of Medicine, The Sahlgrenska Academy at University of Gothenburg, Gothenburg, Sweden; 3 Present Address: Centre for Bone and Arthritis Research, University of Gothenburg, Gothenburg, Sweden; 4 Present Address: Biogen, Cambridge, MA, USA; 5 Present Address: St. Jude Children’s Research Hospital, Memphis, TN USA; 6 Present Address: MD Anderson Cancer Center, Houston, TX USA; 7 Present Address: Bethyl Laboratories, Montgomery, TX USA; 8 Present Address: Merck, Rahway, NJ USA; 9 Present Address: Nurix, San Francisco, CA USA; 10 Present Address: Wntrix, Houston, TX USA; 11 Present Address: PRA Health Sciences, Raleigh, NC USA; 12 Present Address: University of Pennsylvania, Philadelphia, PA USA; 13 Present Address: Regeneron Pharmaceuticals, Tarrytown, NY USA; 14 Present Address: University of Texas, Houston, TX USA

**Keywords:** Bone, Metabolic disorders

## Abstract

The disability, mortality and costs caused by non-vertebral osteoporotic fractures are enormous. Existing osteoporosis therapies are highly effective at reducing vertebral but not non-vertebral fractures. Cortical bone is a major determinant of non-vertebral bone strength. To identify novel osteoporosis drug targets, we phenotyped cortical bone of 3 366 viable mouse strains with global knockouts of druggable genes. Cortical bone thickness was substantially elevated in *Notum*^*−/−*^ mice. NOTUM is a secreted WNT lipase and we observed high NOTUM expression in cortical bone and osteoblasts but not osteoclasts. Three orally active small molecules and a neutralizing antibody inhibiting NOTUM lipase activity were developed. They increased cortical bone thickness and strength at multiple skeletal sites in both gonadal intact and ovariectomized rodents by stimulating endocortical bone formation. Thus, inhibition of NOTUM activity is a potential novel anabolic therapy for strengthening cortical bone and preventing non-vertebral fractures.

## Bone Development: NOTUM inhibition stimulates bone formation

NOTUM is an enzyme that inactivates WNT proteins (which play a key role in early tissue development), and inhibiting NOTUM has been found to increase the formation of endocortical bone (within the cortex, the hard exterior of bone) and enhance bone strength. Existing therapies for osteoporosis (condition causing bone to become weak and brittle) are effective in reducing vertebral, but not non-vertebral, fractures. A team headed by Robert Brommage at Lexicon Pharmaceuticals, Texas aimed to identify novel osteoporosis drug targets in mice. Following inhibition of NOTUM activity, the authors observed increased cortical bone thickness and strength at multiple skeletal sites through stimulation of endocortical bone formation. The team concluded that inhibiting NOTUM activity has good potential as a new therapeutic strategy and could be beneficial in preventing non-vertebral osteoporotic fractures.

## Introduction

Musculoskeletal diseases are common causes of severe pain and physical disability and their prevalence will increase with the aging of society.^1^ One of the greatest burdens is attributed to osteoporotic fractures, which have an incidence that increases exponentially with age. Although substantial progress has been made in the therapeutic reduction of vertebral fracture risk in osteoporotic individuals, the risk of non-vertebral fractures, which make a greater contribution to mortality,^2,3^ is reduced only marginally by currently available treatments, defining an unmet medical need.^4^ Cortical bone, comprising 80% of skeletal mass, is a major determinant of bone strength and non-vertebral fracture susceptibility, and marrow cavity expansion from endocortical bone loss with age is a major contributor to osteoporosis.^5–10^ Currently used anti-resorptive drugs reduce the risk of vertebral fractures by up to 70%, whereas the risk for non-vertebral fractures is only reduced by 20%,^11^ suggesting that trabecular and cortical bone might respond differently to signals involved in the regulation of skeletal homeostasis. The recent identification of *SFRP4* mutations in Pyle’s disease, associated with greatly elevated trabecular but severely reduced cortical bone mass, highlights this differential regulation.^12^ Therefore, new insights into the biology of these bone compartments is of great clinical and therapeutic importance.

Screening gene function in vivo is a powerful approach to discovering novel drug targets.^13,14^ Lexicon performed the largest high-throughput phenotypic screening (HTS) campaign to date (4 656 genes) of gene function in mice to identify novel drug targets for various diseases. Within this program, bone phenotyping assessments included microCT measurements of cortical bone thickness.^15^ The genes examined were enriched in enzymes, receptors and secreted proteins (druggable targets), while genes coding for transcription factors and structural proteins were omitted.^16,17^ Femoral cortical bone thickness was evaluated in 3 366 viable gene knockout mouse lines. Successes of this phenotyping campaign included characterization of mouse skeletal phenotypes with disruptions of *Fam20c*,^18^ *Lrrk1*,^19^ *Sfrp4*,^12^ and *Slc10a7*,^15^ all subsequently shown to mimic homologous human gene mutations responsible for non-lethal Raine syndrome,^20^ osteosclerotic metaphyseal dysplasia,^21^ Pyle’s disease^12^, and skeletal dysplasia,^22^ respectively. Similarly, identification of *Wnt16* as a key regulator of cortical bone mass in mice^23–25^ was followed by recognition that *WNT16* SNPs contribute to human BMD and skeletal fragility.^23^

This phenotyping campaign also identified a novel cortical bone drug target, NOTUM, a lipase that inactivates WNTs by cleaving the palmitoleate moiety essential for Frizzled receptor binding and activation.^26–28^ *Notum*^*−/−*^ mice have normal trabecular bone mass, but elevated cortical bone thickness and strength. We developed three orally active small molecules and one neutralizing antibody that inhibit NOTUM enzymatic activity. Pharmacological inhibition of NOTUM increased endocortical bone formation and cortical bone thickness and strength in well-established rodent osteoporosis disease models.

## Results

### High-throughput phenotypic screening identified high cortical bone thickness in *Notum*^*−*^^*/−*^ mice

**Fig. 1:**
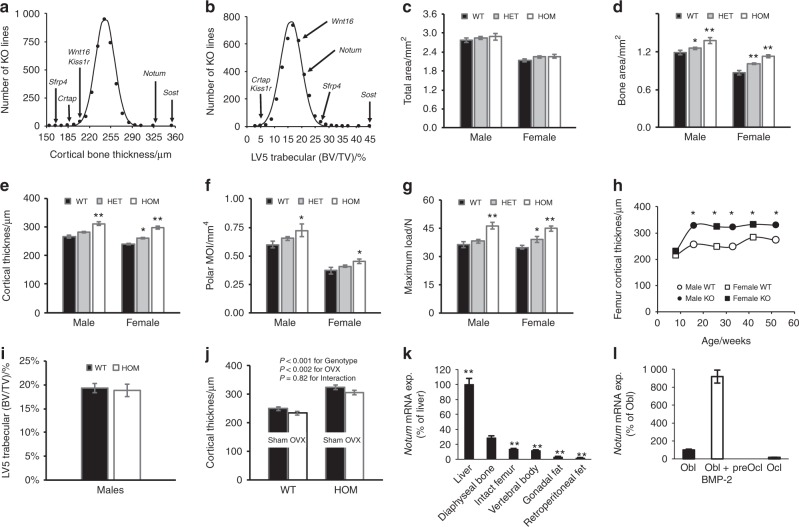
High-throughput phenotypic screening (HTS) identifies high cortical bone thickness in *Notum*^*−/−*^ mice. **a**, **b** Histograms showing distribution of midshaft femur cortical bone thickness of *N* = 13 845 mice from 3 366 distinct global gene knockout mouse lines and trabecular bone volume fraction (BV/TV) in the fifth lumbar vertebral body of *N* = 13 979 mice from 3 399 distinct global gene knockout mouse lines. Mean values are normally distributed. HTS data for selected genes with substantially increased or decreased values are indicated by arrows. **c**–**g** 16-week-old *Notum*^*−*^/^*−*^, *Notum*^*+/*−^ and WT mice. **c**–**f** Midshaft femur cortical total bone area, bone area, cortical thickness, polar MOI (N males: WT = 23, *Notum*^+/− ^= 50, *Notum*^−/− ^= 17; females: WT = 22, *Notum*^+/− ^= 57, *Notum*^−/− ^= 18). **g** Femur shaft bone strength (maximum load; Max. load) measured by 4-point bending (N males: WT = 23, *Notum*^+/− ^= 20, *Notum*^−/− ^= 17; N females: WT = 21, *Notum*^+/− ^= 20, *Notum*^−/− ^= 18). **h** Midshaft femur cortical bone thickness in male and female *Notum*^*−/−*^ and WT mice at different ages (*N* males: WT: 4 weeks = 14, 16 weeks = 10, 32 weeks = 6, 52 weeks = 6; N *Notum*^−/−^: 4 weeks = 13, 16 weeks = 10, 32 weeks = 6, 52 weeks = 8; N females: WT: 4 weeks = 9, 16 weeks = 8, 26 weeks = 13, 43 weeks = 13; N *Notum*^−/−^: 4 weeks = 10, 16 weeks = 9, 26 weeks = 14, 43 weeks = 9). **i** Vertebral trabecular BV/TV (central part of vertebral body; LV5) of 16-week-old male mice (N: WT = 23, *Notum*^−/−^ = 17). **j** Effect of ovariectomy (OVX) on midshaft femur cortical bone thickness in WT and *Notum*^*−/−*^ mice. OVX or sham-surgery was performed at 18 weeks of age and bones were analyzed 8 weeks later (N WT: sham = 13, OVX = 13; N *Notum*^−/−^: sham = 14, OVX = 16), lean ^*P* < 0.01 OVX vs sham. **k** *Notum* mRNA levels in different tissues in female mice. Arbitrary levels are given with liver indicated as 100%. **P < 0.01 vs tibia diaphyseal bone without marrow (*N* = 10–11). **i** *Notum* mRNA in primary cultured mouse osteoblasts (Obl), pre-osteoclasts (preOcl) and osteoclasts (Ocl). BMP-2 is indicated for BMP-2 stimulated osteoblasts. Arbitrary levels are given with unstimulated Obl indicated as 100% with four cell cultures per data point

A total of 4 656 druggable genes were selected for global knockout studies using either homologous recombination (64%) or gene trap (36%) technologies. Among these, 3 762 distinct gene KO mouse lines with viable adult homozygous mice were examined by DXA and/or µCT scans with femur cortical thickness data available for 3 366 mouse lines (Figure S1).^15^ Two mouse lines, *Sost*^*−/−*^ and *Notum*^*−/−*^, had ≥ 5 SD higher cortical bone thickness than the mean of all evaluated lines (Fig. 1a). Sclerostin, encoded by *SOST*, is an established osteoporosis drug target with the sclerostin antibody romosozumab showing anti-fracture efficacy in a Phase 3 clinical trial.^29^ NOTUM is a potential osteoporosis drug target.

*Notum* gene disruption increased femur cortical bone thickness (Fig. 1a) but not trabecular BV/TV in vertebrae (Fig. 1b). Since NOTUM is a potential cortical bone specific osteoporosis target, we selected *Notum*^*−/−*^ mice for further analyses. Successful gene targeting was verified by lack of *Notum* expression in *Notum*^*−/−*^ mouse liver (Figure S2). DXA analyses revealed increased body BMD in both male and female *Notum*^*−/−*^ mice (Table 1). Multiple cohorts (a total of 250 WT and 212 *Notum*^*−/−*^ adult mice) of *Notum*^*−/−*^ mice consistently had elevated cortical bone thickness in all bones examined, including femur, tibia, humerus, radius, pelvis and femoral neck in both male and female mice (Fig. 1e, j, Figure S3, Tables S1, S2, S3). Increased femoral cortical bone thickness was accompanied by increased mineralized bone area, polar moment of inertia and bone strength (Fig. 1d, g) but total bone area was unaffected (Fig. 1c). Femur cortical bone thickness was elevated in both male and female *Notum*^*−/−*^ mice by 16 weeks without further increases through one year of age (Fig. 1h). Heterozygous (*Notum*^*+/*−^) mice also had increased body BMD, cortical bone thickness, area and strength compared with WT mice (Table 1, Fig. 1c–g). Femur length was normal in both male and female *Notum*^*−/−*^ mice (Table S3). Trabecular BV/TV in LV5 was unchanged in *Notum*^*−/−*^ mice (Fig. 1i). *Notum*^*−/−*^ mice, but not *Notum*^*+/−*^ mice, had slightly reduced body weight, lean body mass and body fat compared with WT mice (Table 1). Elevated cortical bone thickness in *Notum*^*−/−*^ mice compared with WT mice remained following post-pubertal ovariectomy-induced bone loss (OVX; Fig. 1j), with no significant interaction between genotype and OVX. This observation suggests the pathways through which NOTUM and estrogens affect cortical bone thickness are independent. These studies examining *Notum*^*−/−*^ mice demonstrate that NOTUM is a major regulator of cortical bone thickness and strength.

**Table 1:** DXA analyses of male and female *Notum*^−/−^ mice

Index	WT	*Notum* ^+/−^	*Notum* ^−/−^
*Males*	*N*=23	*N*=52	*N*=19
Body weight/g	35.1±0.9	35.1±0.7	29.6±1.0**
Lean body mass/g	26.8±0.5	26.8±0.3	24.7±0.6**
Fat mass/%	23.2±1.2	22.9±1.0	17.4±1.3**
Body BMD/(mg·cm^−2^)	55.5±0.6	58.0±0.5	58.1±1.2*
Spine BMD/(mg·cm^−2^)	60.7±1.5	66.1±1.2	68.2±2.7*
Femur BMD/(mg·cm^−2^)	77.5±1.4	80.9±0.9	82.1±2.2
*Females*	*N*=22	*N*=57	*N*=18
Body weight/g	26.5±1.5	27.0±0.5	22.3±0.6**
Lean body mass/g	19.9±0.3	20.1±0.2	18.1±0.4**
Fat mass/%	24.1±1.2	24.9±0.9	18.9±0.7**
Body BMD/(mg·cm^−2^)	52.7±0.5	55.2±0.4**	57.1±0.6**
Spine BMD/(mg·cm^−2^)	64.5±1.2	67.7±0.8	71.3±1.2**
Femur BMD/(mg·cm^−2^)	67.3±0.8	70.4±0.6**	74.9±1.1**

Body composition and bone mineral density parameters measured by DXA at 14 weeks of age in *Notum*^−/−^, *Notum*^+/−^ and wild-type (WT) mice. All values are means ± SEM.

*P < 0.05, **P < 0.01 vs wild-type (WT)

We previously described dentin dysplasia and incomplete pre-weaning lethality (32%, *N* = 3 391) in *Notum*^*−/−*^ mice.^30^ Since 26% of surviving *Notum*^*−/−*^ mice examined (*N* = 89) had unilateral kidney agenesis^30^ and WNT signaling is critical during kidney development,^31,32^ we suspect lethality results from bilateral kidney agenesis. Incomplete penetrance of human genetic diseases^33^ and of lethal phenotypes during mouse development^34^ are common. Besides dentin formation defects, histological examination of 40 soft tissues from *Notum*^*−/−*^ mice (*N* = 8) at 40 weeks of age revealed no apparent non-skeletal phenotypes (Table S4). Teeth showed secondary pulpal and periosteal inflammation^30^ associated with increased serum globulin levels and white blood cell counts in adult *Notum*^*−/−*^ mice (Table S5). Histological views of molar abnormalities are provided in Figure S4. Aside from mild anemia, all other clinical chemistry values and blood cell counts were normal (Table S5). The slightly lower body weight, lean body mass and body fat observed in *Notum*^*−/−*^ but not *Notum*^*+/−*^ mice (Table 1) are consistent with mild chronic inflammation. Importantly, increased cortical bone mass and strength were observed in both *Notum*^*−/−*^ mice and *Notum*^*+/−*^ mice compared with wild-type mice (Fig. 1d–g).

We observed *Notum* mRNA expression in several mouse tissues, revealing highest *Notum* expression in bone and liver (Fig. 1k, S5A). The expression of *Notum* mRNA was higher in tibial diaphyseal bone with marrow removed than in vertebral body and intact femur, demonstrating that *Notum* expression is higher in cortical than trabecular bone and marrow (Fig. 1j). *Notum* mRNA levels were high in primary mouse osteoblast cultures, particularly after BMP2 stimulation (Fig. 1l). Similarly, analysis of a published gene expression dataset^35^ showed *Notum* expression was elevated eightfold during BMP2-induced differentiation of cultured C2C12 murine myoblasts into osteoblasts and 14-fold when the myoblasts were previously stably transfected with microRNA-318 (Figure S5B). In contrast, no or very low *Notum* expression was observed in primary mouse pre-osteoclast and mature osteoclast cultures, respectively (Fig. 1k). A human gene expression panel^36^ observed *NOTUM* expression in multiple tissues, with bone not examined.

### Orally active NOTUM inhibitors increase cortical bone thickness and strength

As NOTUM contains a typical alpha/beta-hydrolase domain found in lipases, we examined if NOTUM could hydrolyze the fluorescent lipase substrate OPTS (8-octanoyloxypyrene-1,3,6-trisulfonate) and detected robust lipase activity (Fig. S6A). The inactive NOTUM mutant S232A, which replaces the serine residue in the catalytic triad critical for enzymatic activity,^27^ showed no lipase activity (Fig. S6A, WO2012/071381 and US 2014/0256784 A1 patents). As all WNTs are acylated with palmitoleate,^37^ which is essential for Frizzled receptor binding and signaling,^26^ we tested if NOTUM could inactivate WNTs by functioning as a WNT lipase. Treatment of WNT3A with wild-type NOTUM shifted WNT3A from the detergent to water phase (Fig. S6B), consistent with WNT delipidation. In addition, NOTUM inhibited the cellular actions of human WNT3A measured by both β-catenin degradation (Fig. S6C) and the TCF/LEF CellSensor™ canonical WNT signaling reporter assay (Fig. S6D). Mutant NOTUM was inactive in both assays. These findings agree with those of two independent laboratories.^27,28^

Based on the cortical bone phenotypes observed in *Notum*^*−/−*^ mice and knowing NOTUM is a WNT-inactivating lipase predicted to be secreted extracellularly, we hypothesized that NOTUM inhibition, achievable either by orally active small molecule NOTUM inhibitors or neutralizing antibodies, might increase cortical bone thickness and strength. We therefore employed robust enzymatic (Figure S5A) and cell-based (Figure S5D) assays for medicinal chemistry and neutralizing antibody drug discovery programs. The enzymatic assay was employed for HTS of a chemical library, with the cell-based assay providing confirmation of HTS hits. This screening campaign, followed by medicinal chemistry efforts,^38,39^ identified three promising small molecule NOTUM inhibitors (LP-914822, LP-922056, and LP-935001) that potently inhibited mouse, rat and human NOTUM (Figure S7) and had appropriate pharmacokinetic properties for mouse and rat pharmacology studies.

**Fig. 2:**
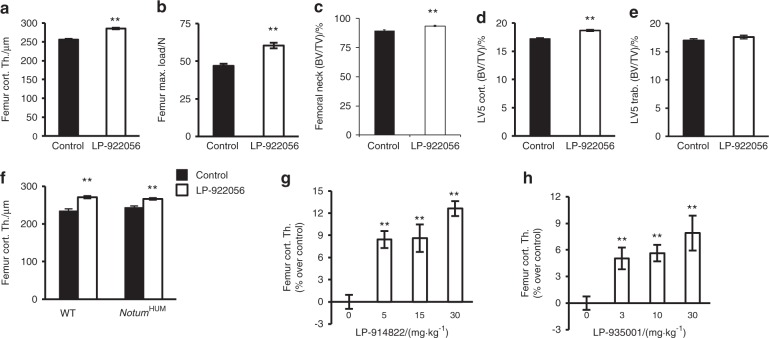
Orally active NOTUM inhibitors increase cortical bone thickness and strength. **a–e** Twelve-week-old male mice were treated with NOTUM inhibitor LP-922056 (*n* = 24; 10 mg·kg^−1^ for 4 weeks by diet) or control diet (*n* = 24). **a** Midshaft femur cortical bone thickness. **b** Femur neck bone volume per total volume (BV/TV; %: mainly reflecting cortical bone). **c** Femur diaphyseal bone strength (maximum load; Max. load) measured by 4-point bending. **d** Vertebral body cortical shell bone volume per total volume (BV/TV; reflecting cortical bone, LV5). **e** Vertebral body trabecular bone volume per total volume (BV/TV; central part of vertebral body; LV5). **f** Femur cortical bone thickness in WT and *NOTUM* humanized male mice (*Notum*^*HUM*^). LP-922056 treatment was given during 4 weeks at a dose of 30 mg·kg^−1^ via diet starting at 12 weeks of age (*N* = 8–9). **g**, **h** Dose-response effects of LP-914822 (doses given twice daily by oral gavage for 25 days to male mice starting at 9 weeks of age, *N* = 13) (**g**) and LP-935001 (doses given by daily oral gavage for 24 days to male mice starting at 13 weeks of age, *N* = 12–16) (**h**) on femur cortical bone thickness given as % increase above vehicle-treated mice. Values are means ± SEM. **P < 0.01 vs controls

Initial dose-response studies in mice using either daily diet administration or twice weekly oral gavage revealed that the small molecule NOTUM inhibitor LP-922056 increased cortical bone thickness in a dose-dependent manner (Figure S8). LP-922056 treatment, given by diet at 10 mg·kg^−1^ for 4 weeks, increased cortical bone thickness and strength in midshaft femur (Fig. 2a, b), bone mass in the femoral neck (Fig. 2c) and vertebral body cortical shell (Fig. 2d), but not vertebral trabecular BV/TV (Fig. 2e). LP-922056 treatment did not increase cortical bone thickness in *Notum*^*−/−*^ mice, demonstrating that its actions were mediated via inhibition of NOTUM activity (Figure S9).

To determine if LP-922056 also inhibits human NOTUM action in vivo, we generated a humanized *NOTUM* mouse line (h*NOTUM* transgenic) expressing human *NOTUM* but not mouse *Notum* (for targeting strategy see Figure S10). Midshaft femur cortical bone thickness was similar in untreated wild-type mice and h*NOTUM* transgenic mice and increased similarly following LP-922056 treatment, demonstrating that LP-922056 is a functional inhibitor of human NOTUM (Fig. 2f). Two other NOTUM inhibitors, LP-914822 and LP-935001, also dose-dependently increased cortical bone thickness in mice (Fig. 2g, h). These data demonstrate that orally active small molecule NOTUM inhibitors increase bone strength in mice via increased cortical bone thickness.

### NOTUM inhibition increases endocortical bone formation

**Fig. 3:**
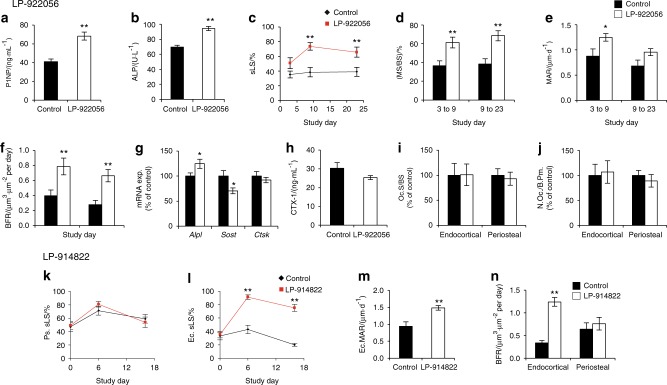
NOTUM inhibition increases endocortical bone formation. **a**–**f** Treatment with NOTUM inhibitor LP-922056. **a**–**b** Male mice were given LP-922056 (10 mg·kg^−1^ per day) by diet administration for 4 weeks. Serum levels of type 1 procollagen N-terminal propeptide (**a**, P1NP, *N* = 24) and alkaline phosphatase (**b**, ALP, *N*: control = 19, LP-922056 = 21) on day 7. **d**–**f** Dynamic histomorphometry of midshaft femur cortical bone. 10-week-old male mice were treated twice weekly with LP-922056 (30 mg·kg^−1^) by oral gavage for 4 weeks. Mice were injected with demeclocycline, alizarin and calcein at days 3, 9 and 23, respectively (*N*: control = 16, LP-922056 = 12). **c** Single-labeled endocortical surface (Ec.sLS). **d** Endocortical mineralized surface per bone surface (Ec.MS/BS). **e** Endocortical mineral apposition rate (Ec.MAR). **f** Endocortical bone formation rate (Ec.BFR). **g** Female mice treated with LP-922056 by daily oral gavage for one week. mRNA levels of *Alp*, *Sost* and *Ctsk* in tibia shaft cortical bone from vehicle (control, *N* = 10) and LP-922056 (30 mg·kg^−1^; *N* = 9) mice. **h**–**j** Female mice treated by daily oral gavage (control, *N* = 8) and LP-922056 (30 mg·kg^−1^; *N* = 8) for 4 weeks. **h** Serum levels of type I collagen C-terminal degradation fragments (CTX-I), **i**, **j** Static histomorphometry of osteoclasts in the midshaft femur. Osteoclast surface per bone surface (**i**, Oc.S/BS; control values were (2.3 ± 0.6)% and (23.7 ± 3.1)% for endocortical and periosteal surfaces, respectively) and number of osteoclasts per bone perimeter (**j**, N.Oc./B.Pm.; control values were (0.95 ± 0.21)  mm^−1^ and (7.94 ± 0.80)  mm^−1^ for endocortical and periosteal surfaces, respectively) expressed as % of control. **k**–**n** Treatment with NOTUM inhibitor LP-914822 (*N* = 13). Male mice were given LP-914822 (30 mg·kg^−1^) by twice daily oral gavage for 25 days. The mice were injected with demeclocycline, alizarin and calcein at days 0, 6 and 16, respectively. **k** Single labeled periosteal surface (Ps.sLS), **l** Single labeled endocortical surface (Ec.sLS). **m** Endocortical mineral apposition rate for days 6 to 16 (Ec.MAR). **n** Endocortical and periosteal bone formation rates for days 6 to 16 (BFR). Values are means ± SEM. *P < 0.05 and **P < 0.01 vs control

We next evaluated the mechanism by which NOTUM inhibition increased cortical bone thickness. NOTUM inhibitor LP-922056 treatment increased serum levels of the bone formation markers procollagen type 1 N-terminal peptide (P1NP; Fig. 3a) and alkaline phosphatase (ALP; Fig. 3b), suggesting enhanced bone formation. This anabolic effect of NOTUM inhibition was supported by observations that LP-922056 treatment increased cortical bone *Alpl* mRNA levels while reducing mRNA levels of the WNT-signaling inhibitor *Sost* (Fig. 3g). Importantly, dynamic histomorphometry revealed that NOTUM inhibition substantially increased endocortical bone formation rate (BFR) as a result of both increased mineralized surface per bone surface (MS/BS) and mineral apposition rate (MAR, Fig. 3c–f). In contrast, NOTUM inhibition did not affect bone resorption as demonstrated by unchanged serum levels of the bone resorption marker CTX-I (C-terminal type I collagen crosslinks; Fig. 3h), cortical bone mRNA levels of the osteoclast-specific transcript *Ctsk* (Fig. 3g) and osteoclast surface per bone perimeter at the endocortical and periosteal surfaces of cortical bone (Fig. 3i, j). A similar effect of NOTUM inhibition on endocortical, but not periosteal, bone formation was observed following treatment with the NOTUM inhibitor LP-914822, which increased midshaft femur cortical thickness from (238 ± 3) to (263 ± 3) µm (*P* < 0.001) (Fig. 3k–n).

### NOTUM inhibition increases mineralization in osteoblast cell cultures

**Fig. 4:**
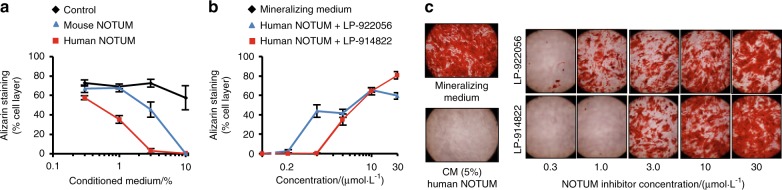
NOTUM inhibition increases mineralization in osteoblast cell cultures. **a** Effect of human and mouse NOTUM conditioned media (CM) on osteoblast mineralization (alizarin red staining) in MC3T3-E1 cells (100% human NOTUM CM = 1.2 μg·mL^−1^; 100% mouse NOTUM CM = 0.4 μg·mL^−1^) (*N* = 3 cell cultures per data point). **b**, **c** Inhibition of mineralization caused by NOTUM (5% human NOTUM CM) was dose-dependently blocked by addition of NOTUM inhibitors LP-922056 or LP-914822 to the culture medium. Both studies lasted 21 days with NOTUM CM present throughout the study. Mineralizing medium = medium without addition of NOTUM CM. Values are means ± SEM of six cell cultures per data point

Exposure of differentiating mouse MC3T3-E1 osteoblast-like cells to conditioned media containing human or mouse NOTUM dose-dependently inhibited mineralization determined by alizarin red staining (Fig. 4a). This inhibition was dose-dependently blocked by addition of the NOTUM inhibitors LP-914822 or LP-922056 to the culture medium (Fig. 4b, c).

### Treatment with orally active small molecule NOTUM inhibitor increases cortical bone mass and strength in ovariectomized (OVX) rats

**Fig. 5:**
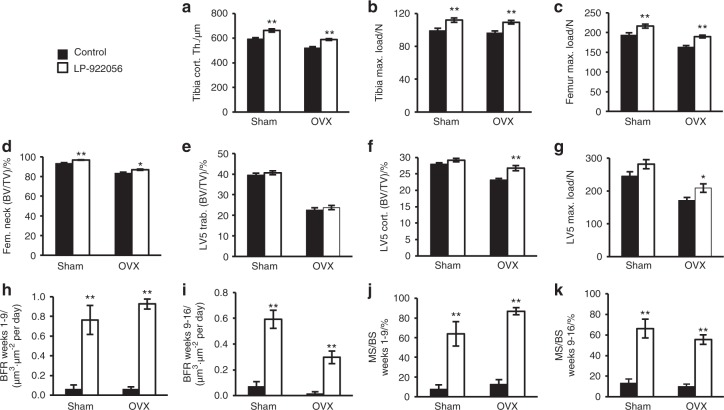
Treatment with orally active small molecule NOTUM inhibitor increases cortical bone thickness and strength in ovariectomized rats. Female rats were ovariectomized (OVX) or sham operated at 21 weeks of age and 18 weeks of treatment with NOTUM inhibitor LP-922056 (daily oral gavage; 30 mg·kg^−1^ per day), or vehicle control was initiated 37 weeks later (*N* = 12–13). **a** Time course of total femur BMD analyzed by DXA throughout the study (*P* < 0.05 for LP-922056 vs control for both gonadal intact and OVX rats at 6, 12, and 18 weeks of treatment), **b** Distal tibia cortical thickness. **c**, **d** Midshaft bone strength in tibia (**c**) and femur (**d**) (maximum load; Max. load) as measured by 3-point bending. **e** Femur neck bone volume per total volume (BV/TV; mainly reflecting cortical bone). **f** Vertebral trabecular bone volume per total volume (BV/TV; central part of vertebral body; L_5_). **g** Vertebral body cortical shell bone volume per total volume (BV/TV; reflecting cortical bone, L_5_). **h** Vertebral compression bone strength (maximum load; Max. Load; LV5). **i**–**l** Dynamic histomorphometry of distal tibia cortical bone (*N* = 6). Rats were injected with alizarin, demeclocycline and calcein at weeks 1, 9, and 16, respectively. Endocortical BFR during weeks 1 to 9 (**i**) and weeks 9 to 16 (**j**) and corresponding endocortical MS values (**k** and **l**) are shown. Values are means ± SEM. *P < 0.05 and **P < 0.01 vs control

**Fig. 6:**
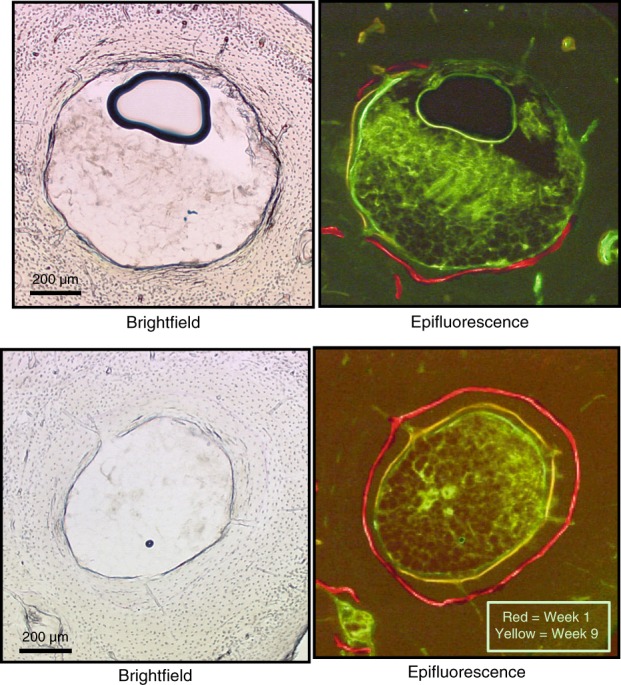
Visualization of endocortical bone formation in ovariectomized rat distal tibia during NOTUM inhibitor treatment. Brightfield (left) and epifluorescence (right) images showing minimal fluorochrome incorporation adjacent to the marrow cavity (center) in control bones (left) but robust endocortical bone formation with NOTUM inhibition (right). Alizarin (red), demeclocycline (yellow), and calcein (green) fluorochromes were given to mice at weeks 1, 9, and 16 during 18 weeks of treatment. Numerical values for this study are provided in Fig. 5

To evaluate the effect of orally active small molecule NOTUM inhibition on bone mass in an established osteoporosis disease model, we studied OVX rats starting at 14 months of age. LP-922056 treatment increased whole femur BMD as measured by DXA in both gonadal intact and OVX rats after 6, 12, and 18 weeks of treatment (Figure S11). In both gonadal intact and OVX rats, LP-922056 treatment increased femoral and tibial cortical bone thickness and strength, and femoral neck BV/TV (Fig. 5a–d). In addition, cortical shell thickness and compression strength but not trabecular BV/TV of the vertebral body were increased by LP-922056 treatment in OVX rats (Fig. 5e–g). A summary of the actions of LP-922056 treatment on various skeletal sites is provided in Figure S12. Dynamic histomorphometry of the midshaft femur and rib (data not shown) and distal tibia diaphysis (Fig. 5i, j) demonstrated that LP-922056 treatment increased cortical bone thickness via substantially increased endocortical BFR (Fig. 5h, i) and mineralized surface (Fig. 5j, k) in both gonadal intact and OVX rats observed both early (weeks 1–9) and late (weeks 9–16) after initiation of treatment. Representative images of greatly elevated fluorochrome labeling at the distal tibia endocortical surface are shown in Fig. 6. Static histomorphometry of LV4 trabecular bone showed expected bone loss with OVX but no effects of LP-922056 treatment on BV/TV, trabecular number and trabecular thickness. Bone remodeling, determined from osteoblast, osteoclast, and osteoid surface measurements, was low and unaffected by LP-922056 treatment (Table S6).

### NOTUM antibody treatment increases cortical bone mass in both gonadal intact and ovariectomized mice

**Fig. 7:**
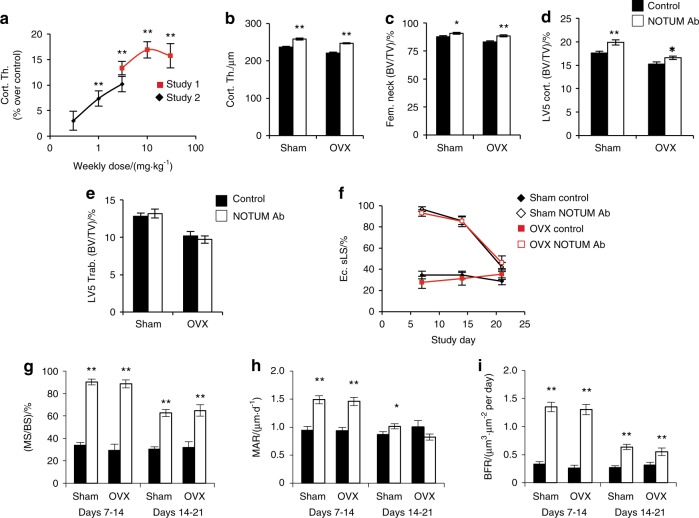
NOTUM antibody treatment increases cortical bone mass in both gonadal intact and ovariectomized mice. **a** In a pilot study, NOTUM antibody 2.78.33 treatment given weekly at 30 mg·kg^−1^ for 8 weeks increased midshaft femur cortical bone thickness in male mice (8-week-old) by (16.4 ± 1.3)% over vehicle-treated mice (*P* < 0.001, not shown in the figure). Two dose-response studies [study 1 (*N* = 10) and study 2 (*N* = 12) as shown in **a**], revealed an optimal dose of 10 mg·kg^−1^ given by intraperitoneal injection once weekly for 4 weeks to 8-week-old male mice. Effects on femur cortical bone thickness are shown as percent increases compared to control antibody treated mice. **b**–**j** Effects of NOTUM antibody treatment on bone parameters in sham-operated and ovariectomized (OVX at 16 weeks of age) mice. At 24 weeks, four weekly injections of NOTUM neutralizing antibody 2.78.33 (10 mg·kg^−1^ intraperitoneal injection) or control antibody for were initiated. **b** Midshaft femur cortical thickness. **c** Femoral neck bone volume per total volume (BV/TV; mainly reflecting cortical bone). **d** LV5 vertebral cortical shell BV/TV; reflecting cortical bone. **e** LV5 vertebral body trabecular BV/TV. **f**–**j** Dynamic histomorphometry of midshaft femur cortical bone. Mice were injected with calcein, alizarin and demeclocycline on days 7, 14, and 21, respectively. **f** Single-labeled endocortical surface (Ec.sLC). **g** Endocortical mineralized surface per bone surface (MS/BS). **h** Endocortical mineral apposition rate (MAR). **I** Endocortical bone formation rate (BFR). Values are means ± SEM (*N* = 11–14), *P < 0.05 and **P < 0.01 vs control

As a second approach to achieve effective NOTUM inhibition, we generated neutralizing monoclonal mouse anti-NOTUM antibodies. Antibody 2.78.33 had IC50 potencies of 37 nmol·L^−1^ and 4 nmol·L^−1^ in enzymatic and cell-based assays, respectively, and was selected for mouse studies. Dose-response studies assessing the effect of Ab 2.78.33 on cortical bone thickness in the femur shaft revealed an optimal dose of 10 mg·kg^−1^ given once weekly (Fig. 7a). We next evaluated the effect of treatment with Ab 2.78.33 for four weeks in both sham-operated and OVX mice (Fig. 7b–i). This treatment increased cortical bone thickness (midshaft femur, femoral neck and vertebral body shell; Fig. 7b–d) but not vertebral trabecular BV/TV (Fig. 7e). NOTUM antibody treatment increased cortical bone thickness via a substantially increased BFR at the endocortical bone surface as a result of both increased MS/BS and increased MAR (Fig. 7f–j). The most pronounced increase in endocortiocal BFR was observed between days 7 and 14 after initiation of treatment but was still significantly increased between days 14 and 21 (Fig. 7i). This transient stimulation of endocortical bone formation is consistent with the stable elevation in cortical bone thickness observed in adult *Notum*^*−/−*^ mice (Fig. 1f, g). Representative dynamic histomorphometric images showing endocortical bone formation with NOTUM inhibition are shown in Figure S13. A separate experiment showed that Ab 2.78.33 treatment increased serum levels of the bone formation marker type 1 procollagen N-terminal propeptide (Figure S14).

### Comparisons of NOTUM inhibition to skeletal action of teriparatide

Teriparatide (rhPTH [1–34]), an injectable amino-terminal fragment of parathyroid hormone that stimulates bone formation, has been used for osteoporosis treatment since 2002. In published studies examining six mouse cohorts, we observed a mean increase in midshaft femur cortical thickness of 15% (range of 12% to 17%) with teriparatide treatment at a dose of 80 μg·kg^−1^ (Table S7). Similar efficacies were observed in the present studies examining NOTUM inhibition, with average increases in femur cortical thickness of 18% in male and 21% in female *Notum*^*−/−*^ mice compared with WT mice (Table S1). Femur cortical thickness was increased by 12% (Fig. 2a) and 16% (Fig. 2f) in mice treated with the orally active NOTUM inhibitor LP-922056, and by 16% (Fig. 6a) and 12% (Fig. 6b) in gonadal-intact and OVX mice, respectively, treated with NOTUM antibody 2.78.33.

## Discussion

Cortical bone thickness is a major determinant of fracture risk in humans but few studies have focused on cellular and molecular mechanisms specifically regulating cortical bone mass.^4^ We demonstrated that *Notum*^*−/−*^ mice have increased cortical bone thickness and strength. NOTUM is a member of the alpha/beta-hydrolase superfamily.^40^ Identification of NOTUM’s WNT-inactivating lipase activity and the development of orally active small molecule inhibitors and a neutralizing antibody revealed that NOTUM is a negative regulator of endocortical bone formation and its pharmacological inhibition increases endocortical bone formation and cortical bone strength. As bone formation by osteoblasts is strongly influenced by WNT signaling,^41,42^ NOTUM presumably reduces cortical bone thickness by inactivating crucial WNTs present in the endocortical bone environment.

Our phenotypic screen identified two gene KOs, *Sost*^*−/−*^ and *Notum*^*−/−*^ mice, having ≥ 5 SD increase in cortical bone thickness. Sclerostin, encoded by *Sost*, is an established osteoporosis drug target while NOTUM is a novel osteoporosis drug target. *Sost*^*−/−*^ mice have elevated cortical and trabecular bone mass, while *Notum*^*−/−*^ mice have a specific increase in cortical bone thickness with normal trabecular BV/TV. As previously reported,^15^ our screen also identified four KO mouse lines (*Sfrp4*^*−/−*^, *Crtap*^*−/−*^*, Wnt16*^*−/−*^, and *Kiss1r*^*−/−*^) with substantially reduced cortical bone thickness (Fig. 1a). Two of these lines (*Crtap*^*−/−*^ and *Kiss1r*^*−/−*^) also have reduced trabecular BV/TV while *Sfrp4*^*−/−*^ mice have increased trabecular BV/TV and *Wnt16*^*−/−*^mice have normal trabecular BV/TV. In summary, when evaluating six extremes of low (*Sfrp4*^*−/−*^*, Crtap*^*−/−*^*, Wnt16*^*−/−*^, and *Kiss1r*^*−/−*^) or high (*Sost*^*−/−*^ and *Notum*^*−/−*^) cortical bone thickness, we observed three gene KOs having similar directions of a clear effect on trabecular bone (*Sost*^*−/−*^*, Crtap*^*−/−*^, and *Kiss1r*^*−/−*^), one with opposite effects on trabecular bone (*Sfrp4*^*−/−*^*)* and two having no major effects on trabecular bone (*Notum*^*−/−*^ and *Wnt16*^*−/−*^ (Fig. 1a, b). Collectively, these studies demonstrate that regulation of trabecular and cortical bone mass includes mechanisms having similar, opposite, or neutral effects on these two bone sites. Full understanding of these mechanisms underlying these site-specific skeletal phenotypes remains to be elucidated, but differential gene expression is likely involved.^43^

Bone mass phenotypes identified in gene knockout mice can result from strictly developmental defects, with no skeletal changes occurring if activities of their coded proteins are modulated in adults. Pharmacological inhibition of NOTUM activity in adult mice and rats leading to elevated endocortical bone formation demonstrates that NOTUM has a key role on WNT signaling in bone throughout life. WNT16 also has life-long skeletal actions, as conditional overexpression of human *WNT16* in adult transgenic mice increases cortical bone thickness.^44^

Our mechanistic studies revealed that NOTUM inhibition, using either orally active small molecule NOTUM inhibitors or a neutralizing antibody, increased cortical bone thickness and strength by stimulating modeling-dependent endocortical BFR without significant effects on bone resorption. This elevated endocortical BFR was the result of both increased MS/BS, reflecting the number of active osteoblasts, and MAR, reflecting osteoblast activity. The stimulatory effect of NOTUM inhibition on bone formation is supported by observations that serum levels of the bone formation markers PINP and ALP and cortical bone *Alpl* mRNA expression were increased by NOTUM inhibition. Examination of endocortical bone-forming surfaces after NOTUM inhibition showed smooth fluorescent labels without scalloping resulting from prior osteoclastic resorption. These findings are similar to the anabolic effects of loading^45^ and treatments with PGE2,^46^ teriparatide^47^, and sclerostin antibody,^48,49^ strongly suggesting that NOTUM inhibition activates endocortical bone modeling without affecting bone remodeling.

We observed relatively high *Notum* expression in cortical bone and primary osteoblasts but not in osteoclasts. A direct effect of NOTUM inhibition on osteoblasts is supported by our osteoblast cell culture observations demonstrating that addition of NOTUM suppressed mineralization and pharmacological inhibition of NOTUM activity reversed this suppression. Based on these findings, we propose that bone NOTUM is a crucial local feedback inhibitor of WNT-dependent endocortical bone formation. Future development of conditional *Notum-*inactivated mouse models, targeting different NOTUM expressing cell types, are warranted to identify the cellular source(s) of NOTUM that affect cortical bone.

WNT signaling in tissues involves paracrine actions, with nearby cells communicating information affecting cell differentiation and proliferation. NOTUM binds to cell surface sulfated glycosaminoglycans, facilitating its interactions with WNTs.^27^ NOTUM, although widely expressed,^36^ is able to inactivate WNTs locally and there is no evidence of WNTs acting as systemic hormones. TIKI1 and TIKI2 are metalloproteases that inactivate WNTs by cleaving amino-terminus peptides,^50^ and additional WNT-inactivating enzymes might also exist. Since *NOTUM* expression is upregulated by WNTs,^36^ NOTUM appears to contribute to feedback mechanisms limiting WNT signaling.

Multiple lines of evidence support the potential usefulness of NOTUM as an osteoporosis drug target. First, cortical bone loss in osteoporosis occurs from excessive endocortical bone resorption producing cortical bone trabecularization.^5,7,10^ NOTUM inhibition stimulates endocortical bone formation, thereby increasing cortical bone strength, and might be useful for preventing non-vertebral fractures.^2,3^ Second, as a secreted enzyme that reduces cortical bone thickness NOTUM is a tractable pharmacological target, with orally active small molecule inhibitors or neutralizing antibodies inhibiting its activity and thereby increasing cortical bone thickness. Third, the occurrence of increased bone mass and strength in heterozygous *Notum* mice indicates that NOTUM inhibition does not need to be complete for a therapeutically significant effect. Fourth, we have demonstrated that pharmacologically inhibiting NOTUM activity, using three orally active small molecule inhibitors and one neutralizing antibody, produces anabolic effects on cortical bone thickness and strength in well-established rodent osteoporosis disease models.

As we reported previously, detailed histological examination of adult *Notum*^*−/−*^ mice revealed no apparent non-skeletal phenotypes, except for dentin formation defects during tooth development.^30^ We observed similar dentin defects following pharmacological inhibition of NOTUM in incisor teeth (data not shown), which grow continuously throughout life in rodents. However, dentin defects were not present in non-growing molar teeth of treated adult rodents and dentin formation is minimal in adult humans. Although minimal side effects were observed with pharmacological inhibition of NOTUM, using either orally active small molecules or a neutralizing antibody in adult rodents, further drug development of NOTUM inhibitors is necessary to obtain sufficient therapeutic safety profiles before clinical studies.

Analogs of parathyroid hormone (teriparatide and abaloparatide), given by daily subcutaneous injections, are the currently available anabolic therapies for osteoporosis. The sclerostin antibody romosozumab, given by monthly subcutaneous injections, reduces fractures but regulatory approval has been delayed while cardiovascular safety concerns are evaluated.^51^ In our experience treatments with teriparatide and NOTUM inhibitors (*Notum*^*−/−*^ mice, the orally active small molecule LP-922056 and the neutralizing antibody 2.78.33) are similarly effective in increasing cortical bone thickness, whereas the other anabolic therapies also increase trabecular bone mass. Compared to the clinically used analogs of parathyroid hormone, NOTUM inhibitors have the advantage that they can be given orally and possibly less frequently than daily.

In summary, a promising novel cortical bone osteoporosis drug target, NOTUM, was identified by phenotyping cortical bone thickness of 3 366 mouse strains with knockout of druggable genes. For drug target proof-of-principle, three orally active small molecules and a neutralizing antibody inhibiting NOTUM lipase activity were characterized. They increased cortical bone thickness and strength at multiple skeletal sites in ovariectomized rodents by stimulating endocortical bone formation. Thus, inhibition of NOTUM activity is a potential novel anabolic therapy for strengthening cortical bone and might be useful for preventing non-vertebral osteoporotic fractures.

## Materials and methods

### Gene knockout and high-throughput screening

Gene knockout mice having global disruptions in 4 656 druggable genes were generated using homologous recombination (64%) and gene trap (36%) technologies.^15^ With the exception of 23 genes disrupted in C57BL/6J-derived ES cells, mutations were generated in 129SvEvBrd-derived ES cells used to produce chimeric mice after microinjection into C57BL/6-Tyrc-Brd(albino) blastocysts. Chimeric mice were bred to C57BL/6J albino mice to generate F1 hybrid heterozygous mice, which were intercrossed to generate F2 wild-type (WT), heterozygous and homozygous knockout mutant progeny employed for high-throughput screening (HTS) phenotype analyses. A flow chart of the functional skeletal phenotypic HTS is provided in Figure S1. Femur midshaft cortical thickness of 16-week-old male mice was analyzed for 3 366 distinct KO mouse lines using µCT (averaging 4.1 male KO mice per mutant line; Fig. 1a). For comparison, trabecular bone volume per total volume (BV/TV) in the vertebral body of LV5 was analyzed for 3 399 distinct KO mouse lines (Fig. 1b). *Notum*^*−/−*^ mice (*Notum*^Gt(OST172035)Lex^) were generated by gene trap disruption (OST 172035) of exon 1, determined by inverse PCR, as described previously.^30^

### Mouse husbandry

Lexicon mice were generated and studied within the Lexicon vivarium. Mice were group-housed in micro-isolator cages within a barrier facility at 24 °C on a fixed 12-h light and 12-h dark cycle and fed Purina rodent chow No. 5001 (Purina, St. Louis, MO). Diets and acidified water were provided ad libitum. All procedures involving use of live mice were conducted in conformance with Lexicon’s Institutional Animal Care and Use Committee guidelines that were in compliance with state and federal laws and the standards outlined in the Guide for the Care and Use of Laboratory Animals (National Research Council, 1996). Quarterly sentinel surveillance showed no evidence of pathogenic rodent viruses, *Mycoplasma*, or *Helicobacter* species in the source colonies. All mice and samples were analyzed after investigator blinding to genotypes and experimental treatments. Male C57BL/6J – 129SvEv F1 hybrid mice were examined in pharmacology studies. The OVX studies examined C57BL/6J mice and Fischer 344 rats. Bilateral ovariectomy (OVX) was performed by standard surgical procedures. Mice and rats were fed either standard mouse chow (Purina) or purified 10% low fat diet (D12450, Research Diets, New Brunswick, NJ). In selected studies NOTUM inhibitor compounds were administered by mixing into the purified diet.

### Gothenburg Mice

For mechanistic studies of the effects of NOTUM inhibition on bone formation and bone resorption, twelve-week-old female C57BL/6N mice were treated with LP-922056 (oral gavage, 30 mg·kg^−1^) for one and three weeks at the University of Gothenburg. The stimulatory effect of LP-922056 treatment on cortical bone thickness was confirmed [(12.5 ± 1.9)% over vehicle, *P* < 0.01, *n* = 10]. All mice at Gothenburg were housed in a standard animal facility under controlled temperature (22 °C) and photoperiod (12 h of light, 12 h of dark) and animal care followed institutional guidelines. All animal experiments had been approved by the local Ethical Committees for Animal Research at the University of Gothenburg.

### Recombinant NOTUM and WNT3A

HEK293F cells were stably transfected with human *NOTUM* or S232A-*NOTUM* vector (both with C-terminal His-V5 tags) to provide conditioned media containing active NOTUM or enzymatically inactive S232A-NOTUM (without the essential 232 serine). Purified mouse WNT3A was purchased from R&D Systems (Minneapolis, MN) and mouse WNT3A-conditioned medium was obtained from L Wnt3A cells (ATCC #CRL-2647) grown in DMEM media containing 10% FBS. These cells were grown adherent for 3 to 4 days from a 1:10 to 1:20 split to reach (80–90)% confluency. Cells were replenished with fresh DMEM media containing 10% FBS and cultured for an additional 3 days. Conditioned media were harvested, filtered, and stored at 4 °C.

### NOTUM activity

Enzymatic and cell-based assays of NOTUM activity were employed to screen both a chemical library and antibodies generated against mouse NOTUM. The enzymatic assay was employed for HTS, with the cell-based assay providing confirmation.

#### Enzymatic assay

NOTUM activity was first quantified in an enzymatic assay employing 5 mmol·L^−1^ OPTS (8-octanoyloxypyrene-1,3,6-trisulfonic acid, Sigma, catalog #74875) as the substrate and monitoring the increase in fluorescence (485 nm excitation and 520 nm emission) upon cleavage of the octanoyl group (Figure S5A).

#### Cell-based assay

NOTUM inhibition of WNT activity was quantified in a cell-based TCF/LEF CellSensor® assay having a β-lactamase reporter gene under control of the β-catenin/LEF/TCF response element. β-lactamase activity was measured using a FRET-based substrate (GeneBLAzer®, Invitrogen) technology. LEF/TCF-bla FreeStyle 293F cells (ThermoFischer, catalogue number K1677) were incubated in medium overnight with WNT3A-conditioned medium, NOTUM conditioned medium and either small molecule NOTUM inhibitors or NOTUM neutralizing antibodies (Figure S5D).

Mouse anti-alpha-tubulin, rabbit anti-HA and rabbit anti-ß-catenin antibodies were purchased from Sigma-Aldrich, Invitrogen and Cell Signaling, respectively. The gene encoding CRD-FZD4 was amplified by PCR from a genomic library and, with addition of a C-terminal HA tag, cloned into a pcDNA3+ plasmid before transfection into HEK-293F cells using Lipofectamine™ 2000 (Invitrogen).

### Orally active NOTUM inhibitors

Three small molecule NOTUM inhibitors were examined and full descriptions of chemical syntheses are described separately.^38,39^ Chemical structures and properties are provided in Figure S7. These compounds had excellent oral bioavailability and circulating terminal half-lives of 2.0, 8.3, and 4.8 h for LP-914822, LP-922056, and LP-935001, respectively. Serum levels of these compounds measured during the pharmacology studies are provided in Table S8. Compounds were dosed by oral gavage or by mixture into purified low fat diet, with doses determined knowing both diet drug concentration and food consumption.

### Antibody against NOTUM

Antibodies were raised against purified recombinant mouse NOTUM protein. *Notum*^−/−^ mice were immunized via the hind footpads with a priming immunization of 10 μg mouse NOTUM protein in TiterMax adjuvant with CpG DNA followed by ten boosts of 10 μg mouse NOTUM protein in Alum adjuvant with CpG DNA at three or four day intervals. Inguinal and popliteal lymph nodes were harvested from high titer mice after a final footpad boost with 10 μg mouse NOTUM protein in PBS. Lymph nodes from footpad immunized mice were collected four days after the final boost and were minced and strained to yield a cell suspension. Red blood cells were lysed and the cell suspension was enriched for B-cells by negative selection using magnetic beads coated with antibodies specific for non-B-cell populations. Hybridomas were generated by electro-cell fusion of enriched B-cells with mouse NSl myeloma cells and were seeded onto 96-well plates in hybridoma medium containing hypoxanthine and aminopterin to select for viable B-cell/myeloma cell hybridomas. Hybridomas were screened for the production of NOTUM-specific antibodies by assaying hybridoma conditioned medium for immunoreactivity with anti-6XHis displayed NOTUM protein in an ELISA format. Hundreds of hybridomas secreting antibody specific for mouse and/or human NOTUM were found.

Hybridoma conditioned medium interfered in the OPTS assay, perhaps due to the release from dying cells of hydrolases that could also cleave the OPTS. For this reason, additional hybridoma conditioned medium was generated for those lines originally showing the highest level of binding activity by ELISA and antibody was purified in a 96-well format by affinity chromatography using protein A beads. These purified antibodies were then tested in the OPTS assay at a four-fold dilution without prior quantitation. Antibodies were tested in quadruplicate in 384-well plates. 12.5 μL containing 125 ng of purified NOTUM in 4X reaction buffer (20 mmol·L^−1^ CaCl_2_, 2 mmol·L^−1^ MgCl_2_, 50 mmol·L^−1^ Tris-HCl, pH 7.4) was added to 12.5 μL of purified antibody. After mixing, antibody and NOTUM were incubated at room temperature for 20 min followed by addition of 25 μL of 1.25 μmol·L^−1^ OPTS in 50 mmol·L^−1^ Tris-HCI, pH 7.4. After mixing, the enzyme reaction was allowed to proceed at room temperature for 10 min before being stopped by addition of 25 μL of 3% SDS. Plates were read on an Envision plate reader with an excitation wavelength of 485 nm and emission wavelength of 535 nm to quantify the amount of cleavage product. OPTS assay screening of 1 056 mouse NOTUM immunoreactive hybridomas yielded six antibodies that showed greater than 50% inhibition of mouse NOTUM. These six together with an additional six hybridomas exhibiting some degree of neutralization in the OPTS assay were selected for subcloning by limiting dilution and small scale purified antibody production by protein A affinity chromatography using 50 mL conditioned medium from clonal hybridomas. Antibodies purified from clonal hybridomas were characterized with respect to their species cross-reactivity by ELISA, their ability to recognize reduced, denatured NOTUM protein by Western blot, and their neutralizing potency in the cell-free OPTS assay and the cell-based WNT signaling assay. Twelve monoclonal antibodies neutralized both mouse and human NOTUM in both the OPTS and WNT signaling assays with IC50s in the range of 3–50 nmol·L^−1^. Antibody 2.78.33 (IgG2b, having an enzyme IC50 of 37 nmol·L^−1^ and a cell-based IC50 of 4 nmol·L^−1^) was selected for mouse pharmacology studies. Antibodies were dosed intraperitoneally once per week at the doses indicated.

### Static and dynamic bone histomorphometry

Standard bone histomorphometric measurements were performed using OsteoMeasure software (OsteoMetrics, Decatur, GA, USA) following the guidelines of the American Society for Bone and Mineral Research. For static histomorphometry, femur cortical bone parameters were analyzed by PharmaTest Services, Ltd (Turku, Finland). Briefly, femurs were fixed in 4% paraformaldehyde, dehydrated in 70% ethanol and embedded in methyl methacrylate. After embedding, femurs were sectioned longitudinally and 4-µm-thick sections were stained with Masson-Goldner’s Trichrome. For dynamic histomorphometry, mice and rats were dosed with the fluorochromes calcein (10 mg·kg^−1^, Sigma-Aldrich C0875), demeclocycline HCl (30 mg·kg^−1^, Sigma-Aldrich 1170000), and alizarin complexone (20 mg·kg^−1^, Sigma-Aldrich A3882) to label active bone-forming surfaces. Distal tibia analyses were performed halfway between the tibia-fibular junction and the distal end of the tibia. Bones were embedded in methacrylate and thick sections (~80 μm) prepared with a Leica SP1600 Saw Microtome (Buffalo Grove, IL, USA).^52^

### Biomechanical analyses

Bone strength measurements (maximum load) were performed using an Instron 5500 Mechanical Testing Machine (Norwood, MA) at Numira Biosciences (Salt Lake City, UT). After removal of the posterior pedicle, spinous processes and both cranial and caudal ends with a low-speed diamond saw, LV5 vertebral bodies of rat (4 mm) and mouse (2 mm) were compressed at a rate of 0.6 mm·min^−1^ until failure. Mouse femurs were mounted with a 2.5 mm inner support and a 7.0 mm outer support and 4-point loading was applied at a displacement rate of 3 mm·min^−1^. Strengths of rat femur and tibia shafts were determined from 3-point loading with a 14.0 mm outer support separation and a displacement rate of 6 mm·min^−1^. Femoral neck strength was determined by cantilever compression.

### Biochemical serum bone markers

Serum procollagen type 1 N-terminal propeptide (P1NP) levels were measured using the Rat/Mouse ELISA kit from Immunodiagnostic Systems (IDS, Gaithersburg, MD). Serum alkaline phosphatase (ALP) activity was determined by a clinical chemistry analyzer. As a marker of bone resorption, serum levels of C-terminal type I collagen crosslinks (CTX-I) were assessed using an ELISA RatLaps™ kit (IDS) according to the manufacturer’s instructions.

### Image analyses of the skeleton

A PIXImus DEXA (InsideOutside Sales, Fitchburg, WI) was employed to obtain mouse body and rat femur BMD values.^53^ MicroCT scans of dissected bones were performed with a Scanco µCT40 (Brüttisellen, Switzerland). Scans of cortical shafts involved 20 slices (8 or 16 µm voxel size). Vertebral body cortical and trabecular bone were distinguished as shown in Figure S15. Femoral necks were scanned as previously described,^54^ with representative microCT images shown in Figure S16. We chose to examine trabecular bone in LV5 since the spine has clinical relevance for osteoporotic fractures. Spine trabecular bone mass contributes to vertebral strength and, compared to long bone metaphyses, shows less variability and dependence upon mouse strain, sex and age.

### Gene expression analyses in mouse tissues, primary bone cell cultures, and a differentiating osteoblast cell line

Total RNA was isolated from mouse tissues (liver, tibia diaphyseal bone without bone marrow, vertebral body, intact femur, gonadal fat, spleen, skeletal muscle, heart, interscapular brown fat, and retroperitoneal fat) and primary cells (osteoblasts and osteoclasts) using TRIzol reagent (Sigma) followed by the RNeasy Mini Kit (Qiagen). cDNA synthesis was performed using the High Capacity cDNA Reverse Transcription Kit from Applied Biosystems. Amplifications were performed using the Applied Biosystem StepOnePlus Real-Time PCR System (PE, Applied Biosystems) and Assay-on-Demand primer and probe sets (ThermoFisher Scientific), labeled with the reporter fluorescent dye FAM. Predesigned primers and probes labeled with the reporter fluorescent dye VIC, specific for 18S ribosomal RNA or *Actb*, were included in the reaction as internal standards. Primer identification numbers were: *Notum* (Mm01253273_m1), *Alp* (Mm00475834_m1), *Sost* (Mm00470479_m1), and *Ctsk* (Mm00484036_m1).

*Notum* expression was also determined from a published gene expression database. This study examined BMP2-induced differentiation of murine C2C12 myoblasts into osteoblasts.^21,22^ Stable cell lines expressing control microRNA (C2C12-pMirn0) and microRNA-378 (C2C12-pMirn378) were generated by lentiviral transduction of C2C12 myoblasts. Overexpression of microRNA-378 enhanced *Alpl* expression, mineralization, and mRNA expression of osteogenic marker genes in the presence of BMP2. Cells were plated at 2.5 × 10^4^ cells/cm^2^ (day 1), cultured for 1 day in DMEM 10% NCS, then (day 0) treated with or without 300 ng·mL^−1^ bone morphogenetic protein 2 (BMP2) for 6 days. RNA was extracted on days 0, 3, and 6 and hybridized to GeneChip Mouse Genome 430 2.0 array (Affymetrix) for expression analyses of > 41 000 gene transcripts.

### Effects of NOTUM and NOTUM inhibition on mineralization of MC3T3-E1 cells

MC3T3-E1 cells (ATCC Subclone 4, catalogue # CRL-2593) were seeded at 40 000 cells/16 mm wells in growth medium (αMEM with 10% FBS) for 3 days to reach confluency. Differentiation and mineralization were induced by adding ascorbic acid (50 μg·mL^−1^), β-glycerophosphate (10 mmol·L^−1^) and dexamethasone (100 nmol·L^−1^). Conditioned media from parental HEK-293F cells, a mouse *Notum* HEK-293F line or a human *NOTUM* HEK-293F line were added to the cell medium to examine the effects of NOTUM on differentiation and mineralization. NOTUM inhibition was evaluated by addition of LP-914822 or LP-922056. Cultures were fed every 3 to 4 days with complete change of medium.

### Mouse osteoblast cultures

Primary calvarial osteoblasts were isolated from newborn C57BL/6N mice by sequential digestion of dissected calvaria as described previously.^55^ Primary osteoblasts were cultured for 4–6 days in complete α-MEM medium before subculture in 48-well plates at  40 000 cells/cm^2^ and induction of osteogenic differentiation in complete α-MEM supplemented with 10 mmol·L^−1^ β-glycerophosphate disodium salt hydrate (BGP; Sigma, G9422) and 0.2 mmol·L^−1^ L-ascorbic acid 2-phosphate sesquimagnesium salt hydrate (Asc-2P; Sigma, A8960) with or without 100 ng·mL^−1^ bone morphogenetic protein 2 (BMP-2; R&D Systems, 355-BEC-010) for 7 days. The EMBL-EBI Expression Atlas was employed to search for relevant published mouse *Notum* gene expression data.^56^ This search identified a dataset providing expression data (GeneChip™ Mouse Genome 430 2.0 Array for  45 100 probes) during BMP2-induced differentiation of murine C2C12 myoblasts into osteoblasts.^35^

### Primary mouse osteoclast cultures

Bone marrow cells were flushed from femurs and tibias from 8 to 12 week-old C57BL/6N mice, washed and cultured in complete α-MEM with 30 ng·mL^−1^ M-CSF (R&D, 416-ML-050) in a suspension culture dish (Corning Costar Ins., NY), to which stromal cells and lymphoid cells cannot adhere, at 37 °C for 2–3 days. Cells were washed vigorously with PBS to remove any non-adherent cells and then washed with cold (4 °C) 0.02% EDTA in PBS to release the attached bone marrow macrophages (BMM). BMM purity was demonstrated by expression of the monocyte marker CD11b/Mac-1 in all cells, whereas no cells expressed the T- and B-cells markers CD45R and CD3. Cells were spot seeded in 24-well plates (40 000 cells in 40 μL complete α-MEM). For osteoclast generation, cells were cultured in 30 ng·mL^−1^ M-CSF and 4 ng·mL^−1^ RANKL (R&D, 462-TEC) for 3–4 days.

### Statistical analyses

Numerical data are presented as means ± SEM. Student’s *t*-test was performed between two groups, while ANOVA followed by Dunnett’s test for multiple comparisons was performed when both *Notum*^*−/−*^ and *Notum*^*+/−*^ mice were compared with wild-type mice. Differences were considered statistically significant with *P* < 0.05. The interaction between ovariectomy and genotype (Fig. 1i) was evaluated using the interaction term in a 2-factor ANOVA. All statistical evaluations were two-sided. Sample numbers were determined from power calculations using values from Lexicon’s extensive database of skeletal phenotypes in gene knockout mice.^15^ Extensive data showing reproducibility of *Notum*^*−/−*^ mouse midshaft cortical thickness values (Tables S1 and S2) and the pharmacological actions of LP-914822 (Table S9) are provided.

## Electronic supplementary material

Supplementary Material

## Data Availability

The data that support the findings of this study are available from the corresponding author upon reasonable request. A *Life Sciences Reporting Summary* is available.
