# Supplementary material for: NOTUM inhibition increases endocortical bone formation and bone strength
Source: Bone Res. 2019 Jan 8;7:2. doi: 10.1038/s41413-018-0038-3 (PMC6323125; doi:10.1038/s41413-018-0038-3)
Supplement: Supplementary file 1 — Supplementary Material [file 41413_2018_38_MOESM1_ESM.pdf]

<u>Supplementary Figure 1</u>: High-throughput screening identifies high cortical bone thickness in *Notum*$^{-/-}$ mice

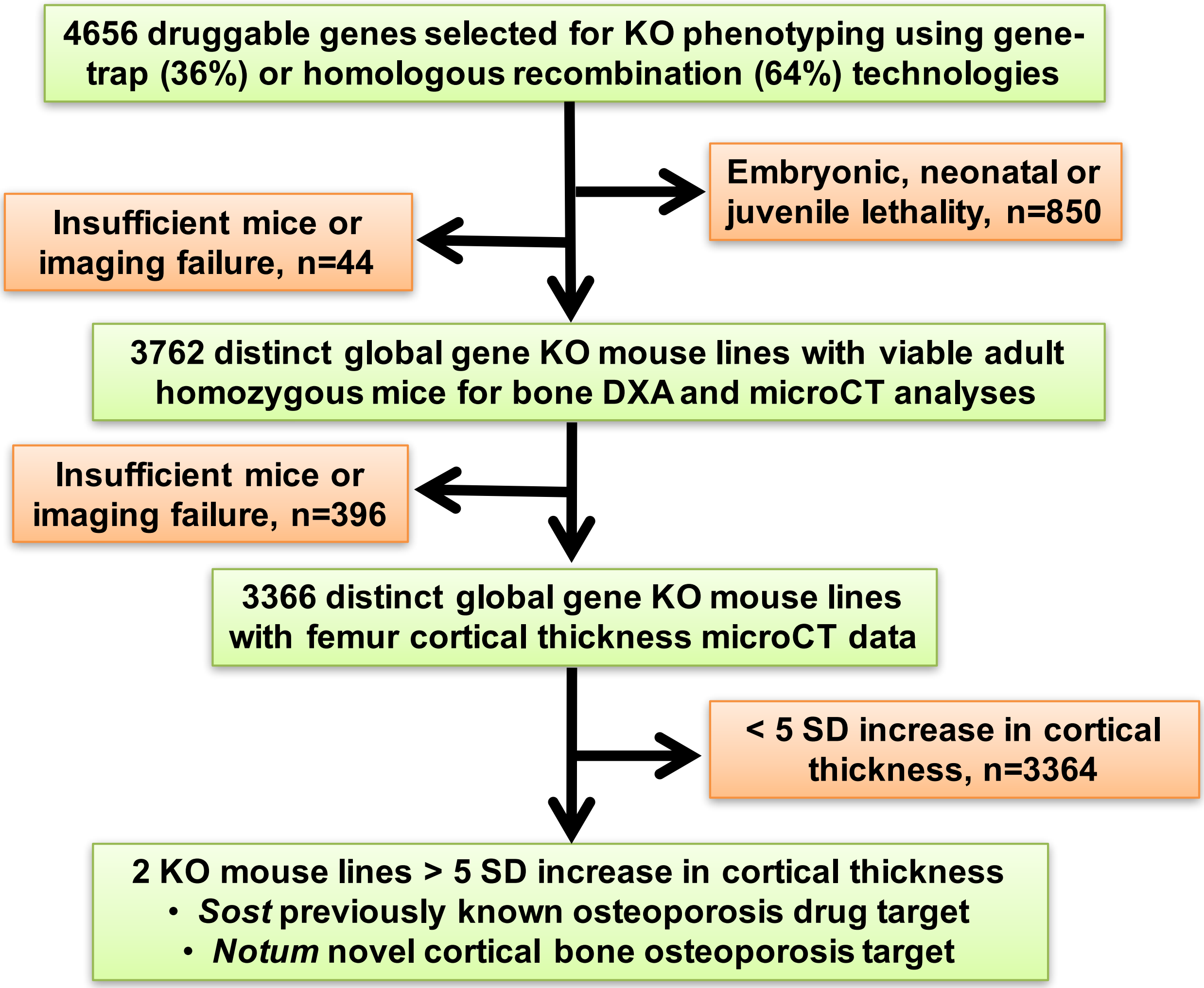

Flow chart of the functional high-throughput phenotypic screening *in vivo* of 4,656 druggable genes, identifying *Notum* as a candidate gene for regulation of cortical bone thickness.

Additional information provided in:

Brommage R, Liu J, Hansen GM, Kirkpatrick LL, Potter DG, Sands AT, Zambrowicz B, Powell DR, Vogel P. High-throughput screening of mouse gene knockouts identifies established and novel skeletal phenotypes. *Bone Res.* 2014; 2:14034.

<u>Supplementary Figure 2</u>: Demonstration of effective gene disruption in *Notum* knockout mice

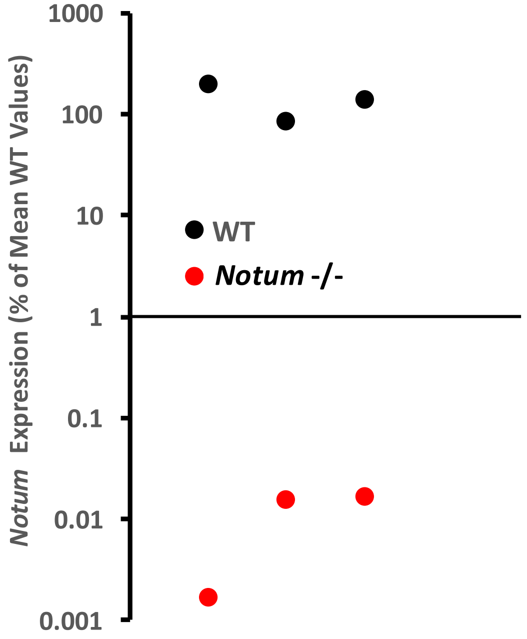

Liver mRNA levels of *Notum* (*Mm01253273_m1*, normalized to *Gapdh*) in *Notum*$^{-/-}$ and wild-type (WT) mice.; N = 3 for each genotype. Gene disruption resulted in 10,000-fold reduction in hepatic *Notum* expression.

Supplementary Figure 3: Representative MicroCT Images of Midshaft Femur Scans

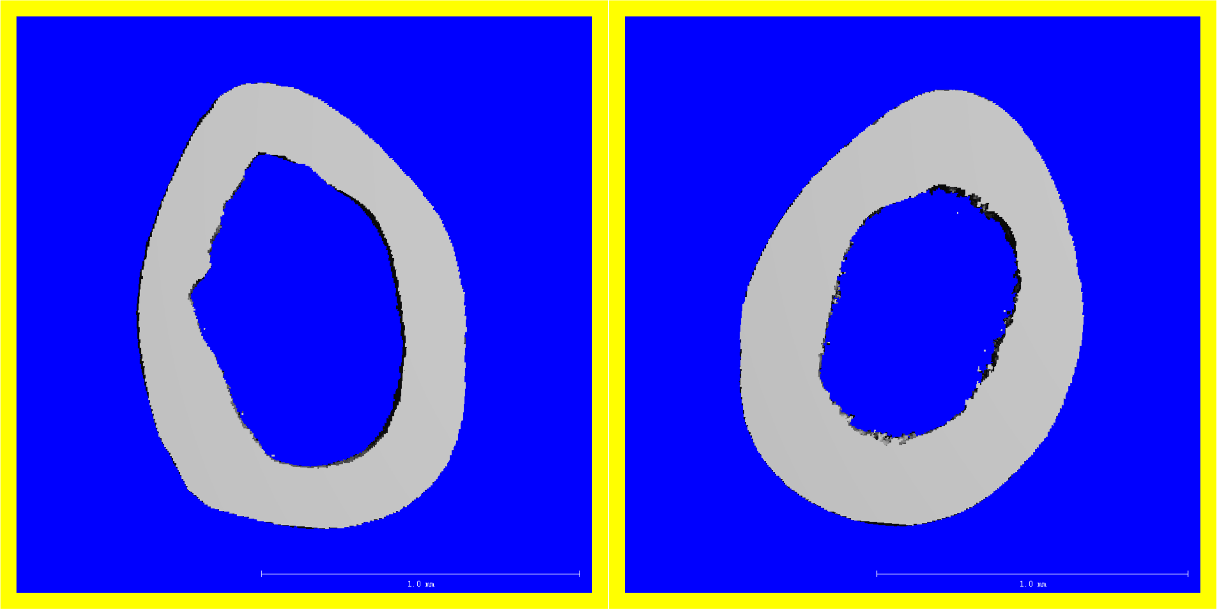

**Ct.Th = 226 µm**	**Ct.Th = 272 µm**
**Mean = 223 µm**	**Mean = 275 µm**

Scans were performed at 6 µm voxel size on femurs from female mice at 16 weeks of age. Mean values are for the full mouse cohorts in the study. This voxel dimension utilizes the maximum resolution of the Scanco µCT 40 scanner.

<u>Supplementary Figure 4A</u>: Cross-Sections Through Molar Teeth from Wild-Type and *Notum*$^{-/-}$ Mice

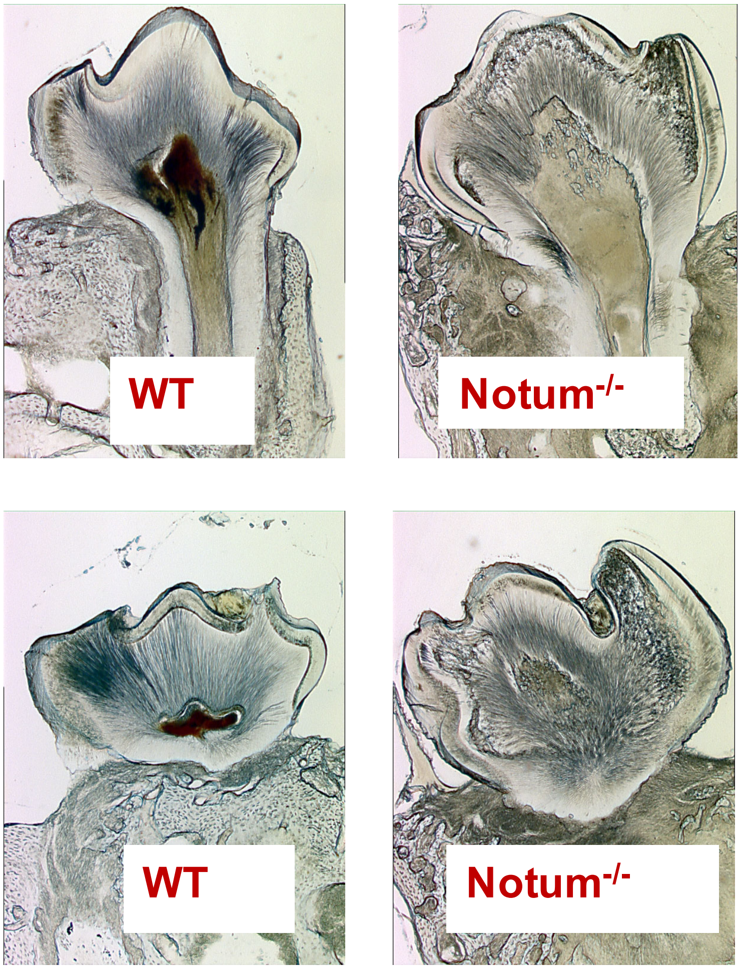

Thick sections of plastic-embedded molar teeth showing abnormal dentin organization. Mice were necropsied at 34 weeks of age.

<u>Further information</u>: Vogel P, Read RW, Hansen GM, Powell DR, Kantaputra PN, Zambrowicz B, Brommage R. Dentin Dysplasia in **Notum** Knockout Mice. Vet Pathol. 2016; 53:853-62.

<u>Supplementary Figure 4B</u>: Frontal sections Through Skulls of Wild-Type and *Notum*$^{-/-}$ Mice

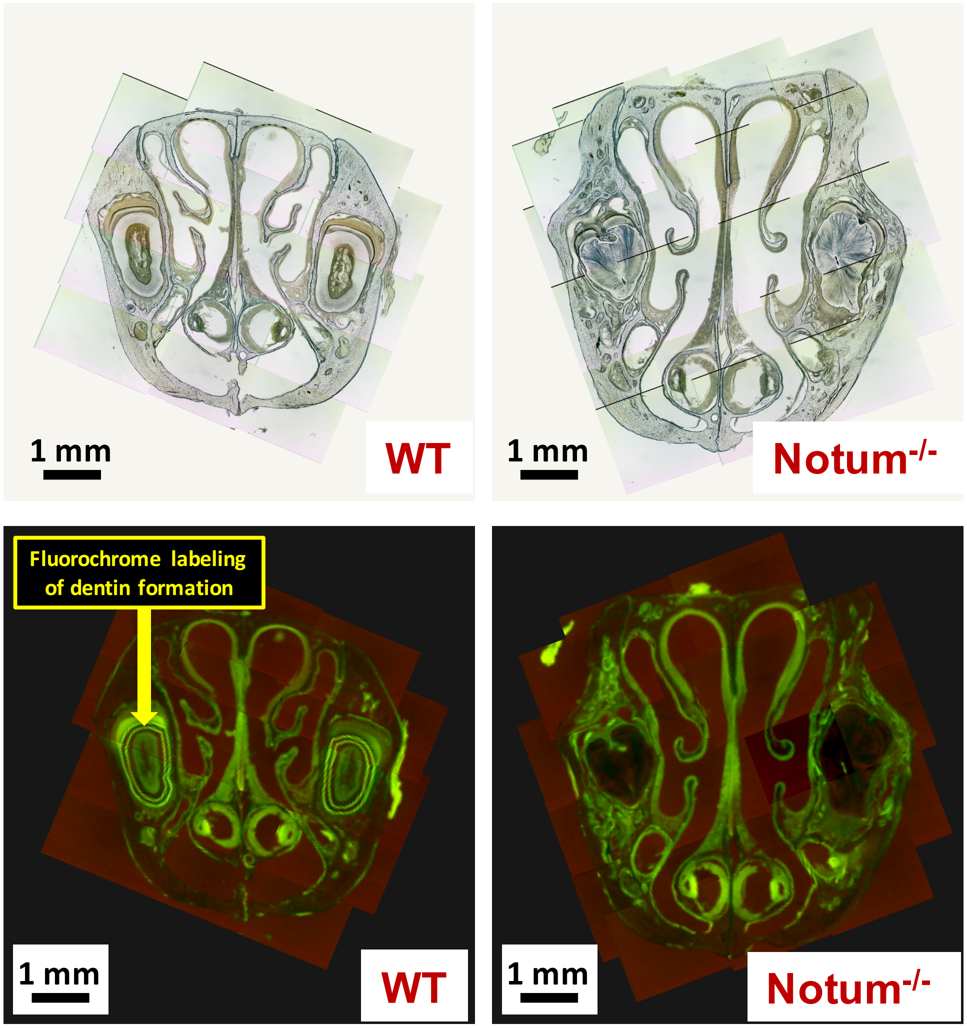

Composite images of thick sections of plastic-embedded molar teeth showing failure of *Notum*$^{-/-}$ mice to incorporate calcein label during dentin formation. Mice were given subcutaneous injections of fluorescent calcein (10 mg/kg) at weeks 32 and 33 of age, with necropsy at 34 weeks.

<u>Supplementary Figure 5</u>: Expression of *Notum* mRNA

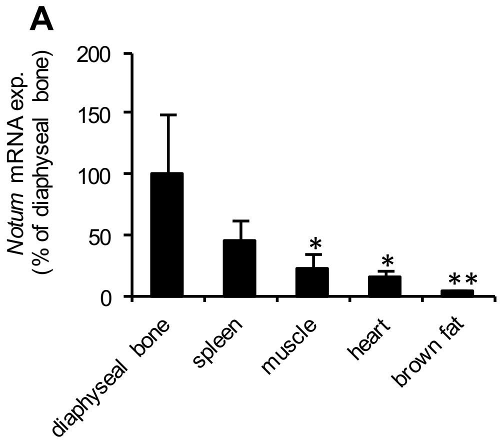

A *Notum* mRNA levels in different tissues of wild-type female mice. Arbitrary levels are given with tibial diaphyseal bone indicated as 100%. ** p< 0.01 vs diaphyseal bone (N = 3-6). Values are means ± SEM. * p< 0.05,** p< 0.01 vs wild-type or diaphyseal bone.

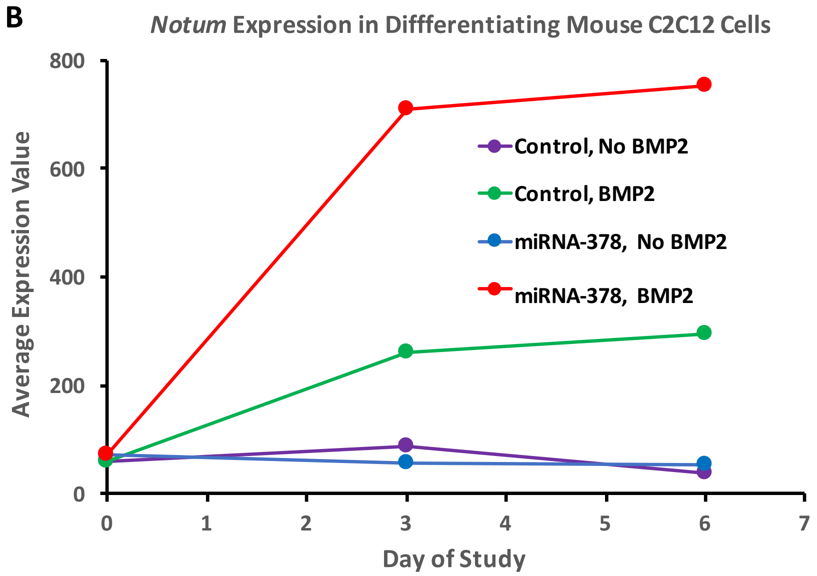

B *Notum* mRNA expression levels during BMP2-induced differentiation of cultured C2C12 murine myoblasts into osteoblasts (Hupkes). Differentiation into osteoblasts and *Notum* expression were enhanced following stable transfection of myoblasts with microRNA-378. Expression values are means of triplicates.

Hupkes M, Sotoca AM, Hendriks JM, van Zoelen EJ, Dechering KJ. MicroRNA miR-378 promotes **BMP2**-induced osteogenic differentiation of mesenchymal progenitor cells. BMC Mol Biol. 2014; 15:1.

Supplementary Figure 6: **NOTUM (but not inactive NOTUM-S232A) exhibits lipase activity, converts lipophilic WNT3A to aqueous soluble WNT3A, and suppresses WNT3A-mediated activation of canonical β-catenin/LEF/TCF signaling pathways in cells**

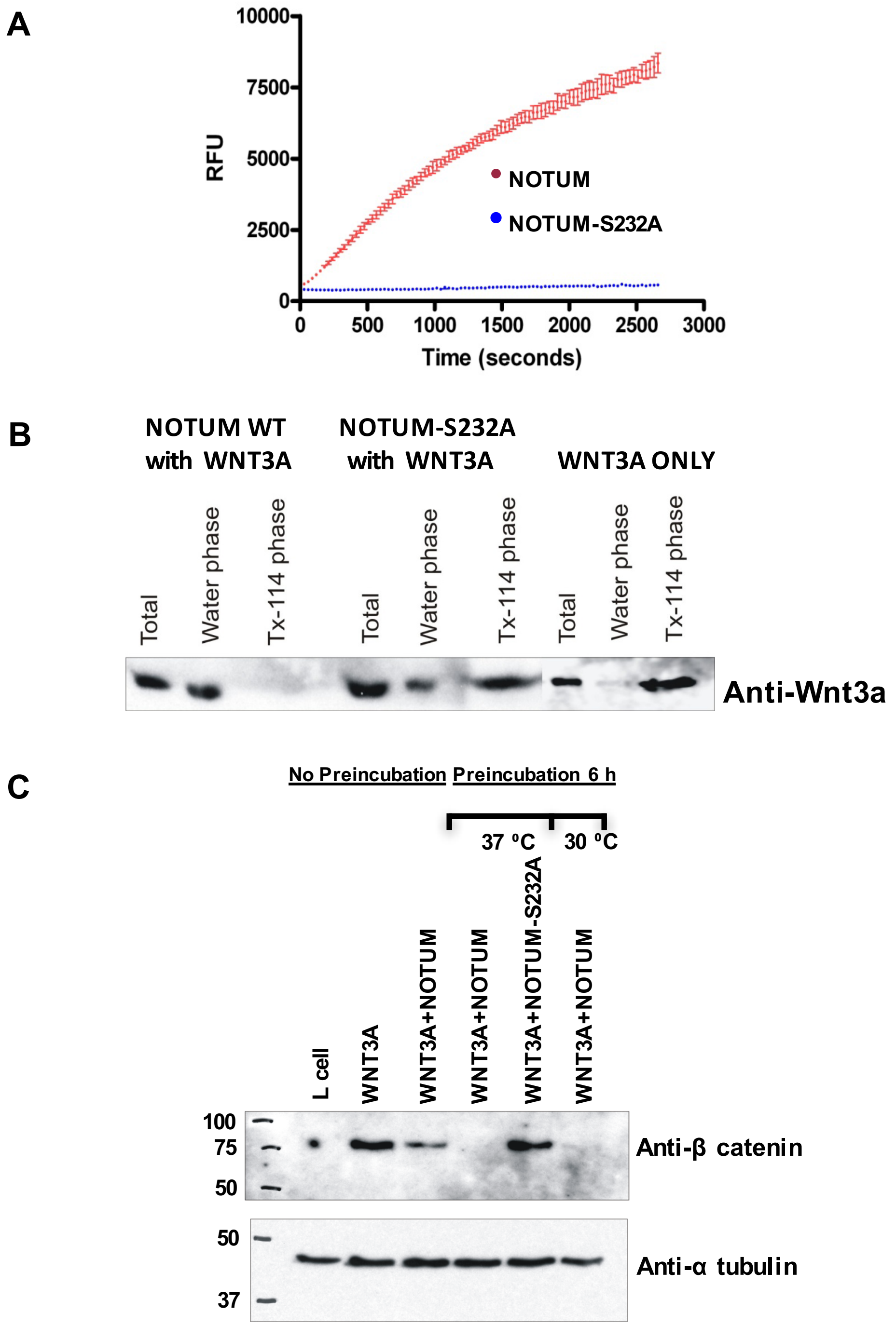

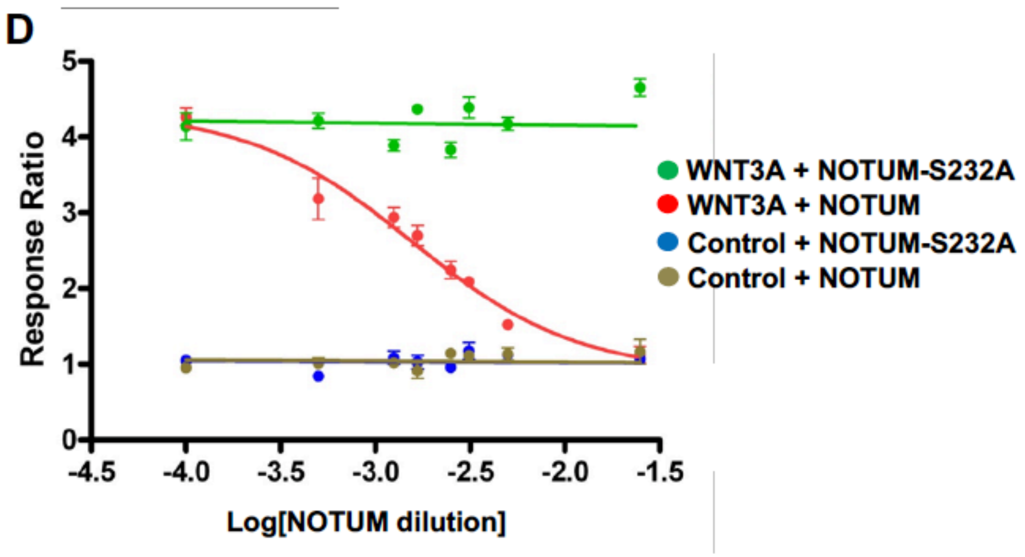

A NOTUM shows lipase activity using OPTS as substrate. The octanoyl moiety of non-fluorescent OPTS is cleaved by NOTUM to yield fluorescent 1-hydroxypyren-3,6,8-trisulfonic acid. Inactive NOTUM mutant S232A shows no lipase activity. RFU = Relative Fluorescence Units.

B NOTUM converts lipophilic WNT3A to aqueous-soluble WNT3A. Mouse WNT3A (200 ng) was incubated with 200 µl human NOTUM conditioned medium at 37 degrees for 6 hours. Triton X-114 detergent was added to 150 µl of samples, followed by incubation on ice for 5 minutes and then at 37 degrees for 5 minutes. Both aqueous and detergent phases, along with untreated WNT3A, were analyzed by Western blotting. Inactive NOTUM mutant S232A shows no activity.

See: Bordier C. Phase separation of integral membrane proteins in Triton **X-114** solution. J Biol Chem. 1981; 256:1604-1607.

C NOTUM disrupts WNT3A-mediated β-catenin stabilization in mouse L cells. First, WNT3A conditioned medium or control conditioned medium were incubated with or without NOTUM or inactive NOTUM-S232A mutant at 37°C or 30°C for 6h. L cells were then stimulated for 2 hours with treated conditioned medium, and cytoplasmic β-catenin was detected by Western blot. Pretreatment of WNT3A conditioned medium with NOTUM but not NOTUM-S232A abolished WNT3A-induced β-catenin accumulation. Co-stimulation of WNT3A and NOTUM reduced cytoplasmic β-catenin levels compared with WNT3A treatment without NOTUM.

D NOTUM abolishes WNT3A-induced LEF/TCF activation detected by the CellSensor™ assay. HEK293 CellSensor cells were co-treated with conditioned medium from WNT3A or control (Control) L cells and with or without NOTUM or NOTUM-S232A overnight. Dose-response effects of NOTUM and NOTUM-S232A on WNT3A-mediated LEF/TCF activity were detected by fluorescent ratio of reporter gene. NOTUM, but not catalytic inactive NOTUM-S232A, blocked WNT3A-induced LEF/TCF transcription factor activation in a dose-dependent manner.

Supplementary Figure 7: Chemical structures and properties of orally-active NOTUM inhibitors examined in rodent pharmacology studies

Property	LP-914822	LP-922056	LP-935001
Molecular Weight (Daltons)	275	301	283
Enzyme IC50 (human)	2 nM	1 nM	0.4 nM
Enzyme IC50 (mouse)	2 nM	1 nM	0.4 nM
Enzyme IC50 (rat)	2 nM	1 nM	NM
Cell-Based IC50 (human)	157 nM	23 nM	12 nM
Cell-Based IC50 (mouse)	643 nM	73 nM	21 nM
Cell-Based IC50 (rat)	309 nM	132 nM	NM
Mouse Plasma Protein Binding	98%	> 98%	90%

NM = Not Measured

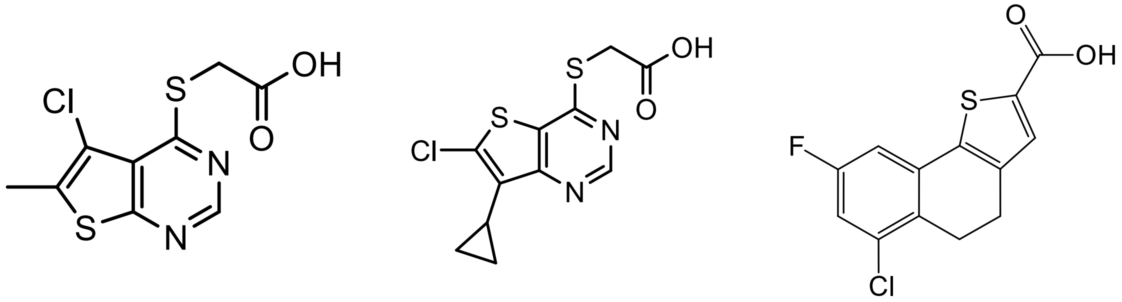

LP-914822 **LP-922056** **LP-935001**

LP-914822: 2-((5-chloro-6-methylthieno[2,3-d]pyrimidin-4-yl)thio)acetic acid

LP-922056: 2-((6-chloro-7-cyclopropythieno[3,2-d]pyrimidin-4-yl)thio)acetic acid

LP-935001: 6-chloro-8-fluoro-4,5-dihydronaphtho[1,2-b]thiophene-2-carboxylic acid

Further information:

Han Q, Pabba PK, Barbosa J, Mabon R, Healy JP, Gardyan MW, Terranova KM, Brommage R, Thompson AY, Schmidt JM, Wilson AG, Xu X, Tarver JE Jr, Carson KG. 4H-Thieno[3,2-c]chromene based inhibitors of Notum Pectinacetylesterase. Bioorg Med Chem Lett. 2016; 26:1184-1187.

Tarver JE Jr, Pabba PK, Barbosa J, Han Q, Gardyan MW, Brommage R, Thompson AY, Schmidt JM, Wilson AGE, He W, Lombardo VK, Carson KG. Stimulation of cortical bone formation with thienopyrimidine based inhibitors of Notum Pectinacetylesterase. Bioorg Med Chem Lett. 2016; 26:1525-1528.

Supplementary Figure 8: Orally active NOTUM inhibitors increase cortical bone thickness and strength

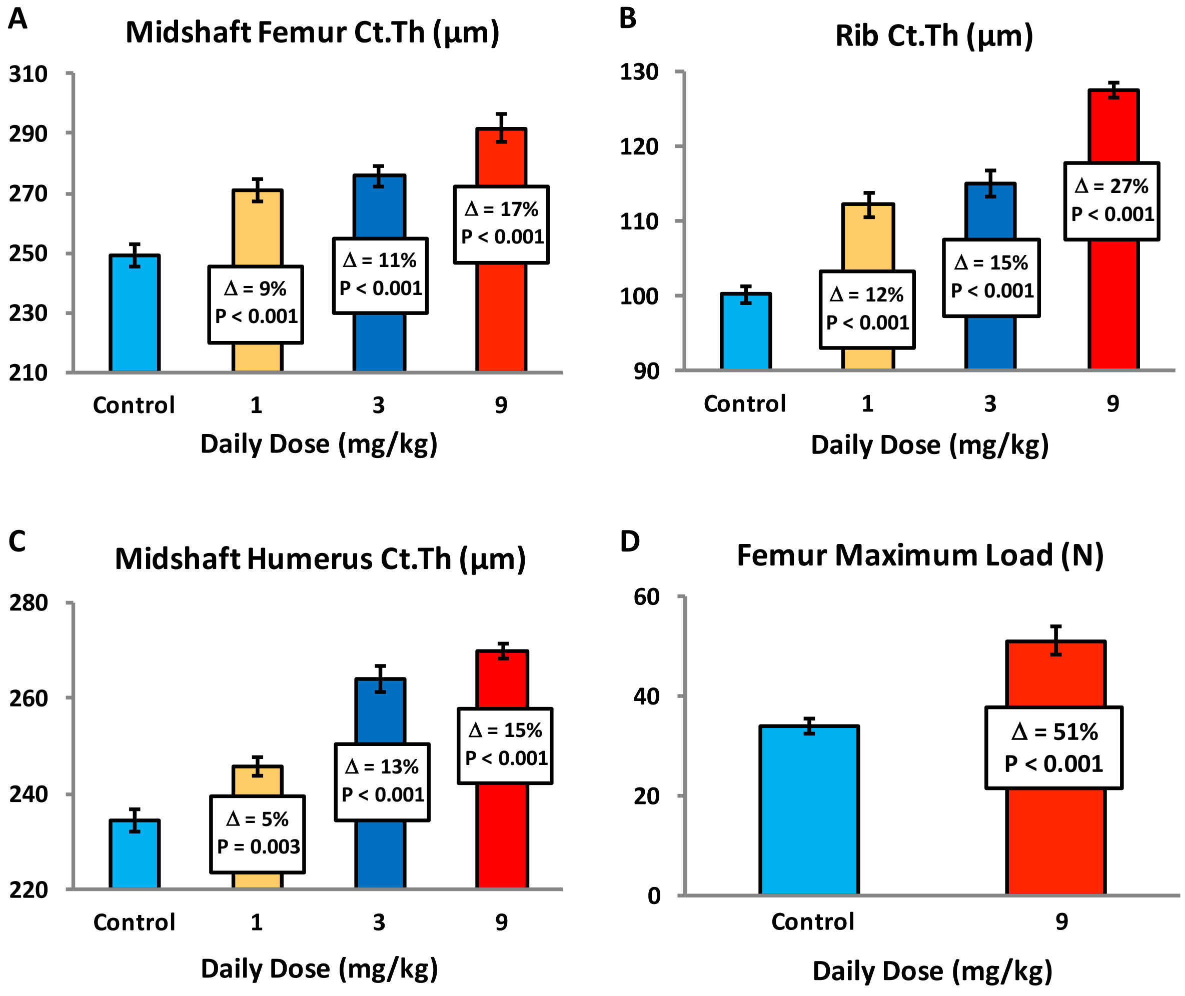

A, B, C, D: Mice were dosed with LP-922056 via diet administration for 12 weeks, starting at 11 weeks of age. There were 15 mice in the control group and 12 mice in each group treated with LP-922056. Data are means ± SEM. For all cortical bone thickness measurements ANOVA $P < 0.001$, with individual Dunnett's P values shown.

<u>Supplementary Figure 8</u>: Orally active NOTUM inhibitors increase cortical bone thickness and strength

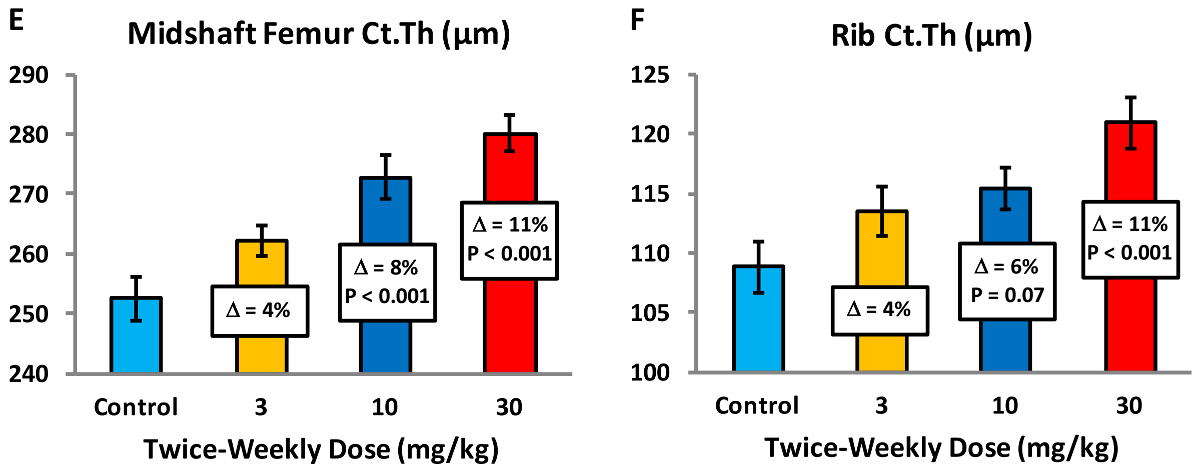

E, F: Mice were dosed Mondays and Thursdays by oral gavage with LP-922056 for 12 weeks, starting at 12 weeks of age. There were 12 mice in each group. Data are means ± SEM, with ANOVA $P < 0.001$ for femur and ANOVA $P < 0.002$ for rib. Individual Dunnett's P values are shown.

<u>Supplementary Figure 9</u>: NOTUM Inhibitor LP-922056 Is Inactive in *Notum*$^{-/-}$ Mice

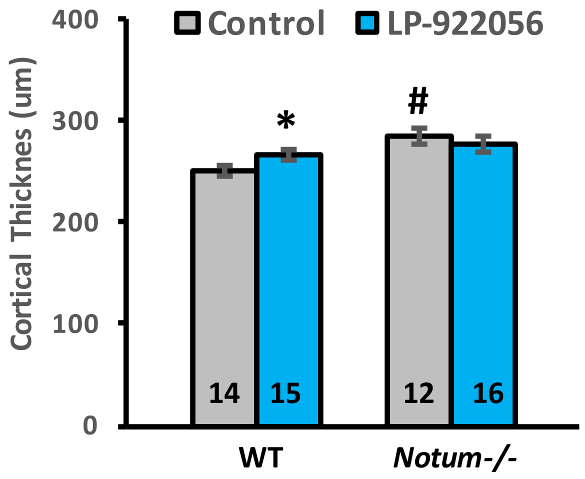

LP-922056 treatment (daily diet administration for four weeks at 30 mg/kg) increased femur cortical bone thickness in 16-week-old female wild type (WT) but not *Notum*$^{-/-}$ mice. All data are means ± SEM for the numbers of mice indicated.

* P < 0.05 compared to untreated WT mice by Student's t-test
# P < 0.01 compared to untreated WT mice by Student's t-test

By two-factor ANOVA,

P < 0.001 for Genotype
P = 0.49 for LP-922056 Treatment
P = 0.04 for Interaction

<u>Supplementary Figure 10</u>: Targeting strategy for *NOTUM* humanized mice

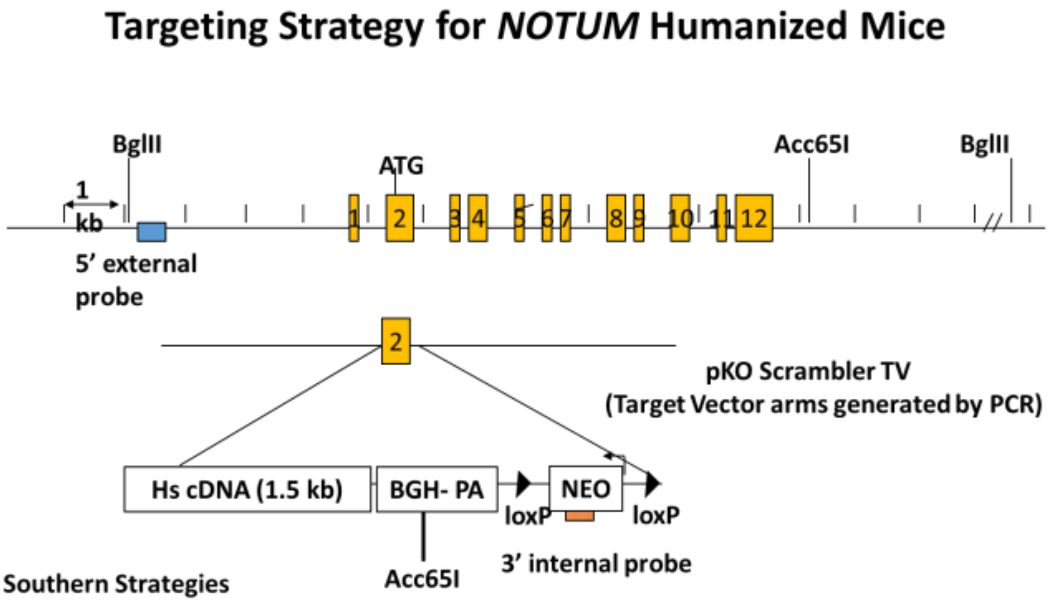

Southern Strategies

Probe	5' external	3' internal
Enzyme	Bgl II	Acc65I
Wildtype	11.7 kb	
Targeted	15.3 kb	7.2 kb

Supplementary Figure 11: Total femur DXA BMD values during treatment of gonadal intact and OVX rats with LP-922056

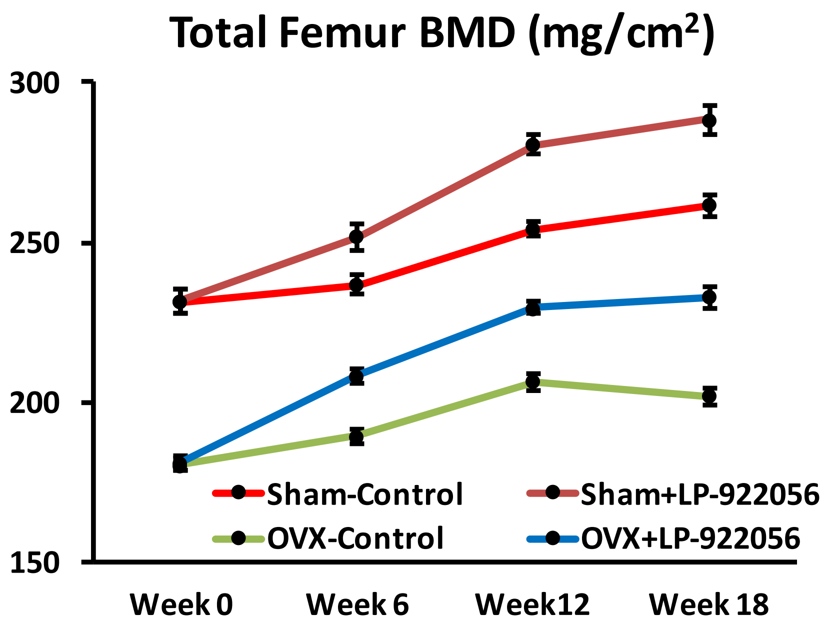

Data are means ± SEM for 12 or 13 rats per group.

<u>Supplementary Figure 12</u>: **Summary of cortical bone changes at various skeletal sites resulting from LP-922056 treatment in gonadal intact and OVX rats**

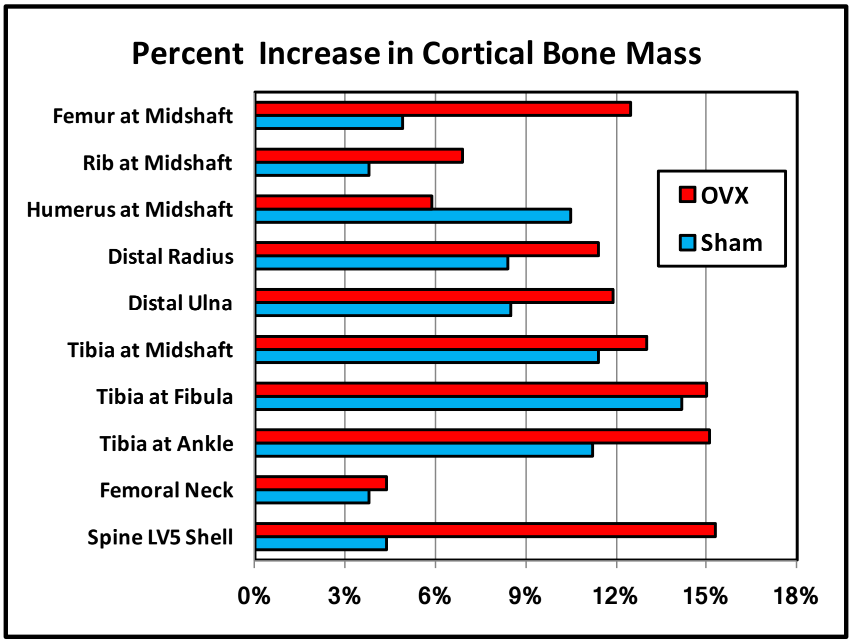

<u>Supplementary Figure 13</u>: Visualization of endocortical bone formation in ovariectomized mouse midshaft femur during NOTUM antibody 2.78.33 treatment

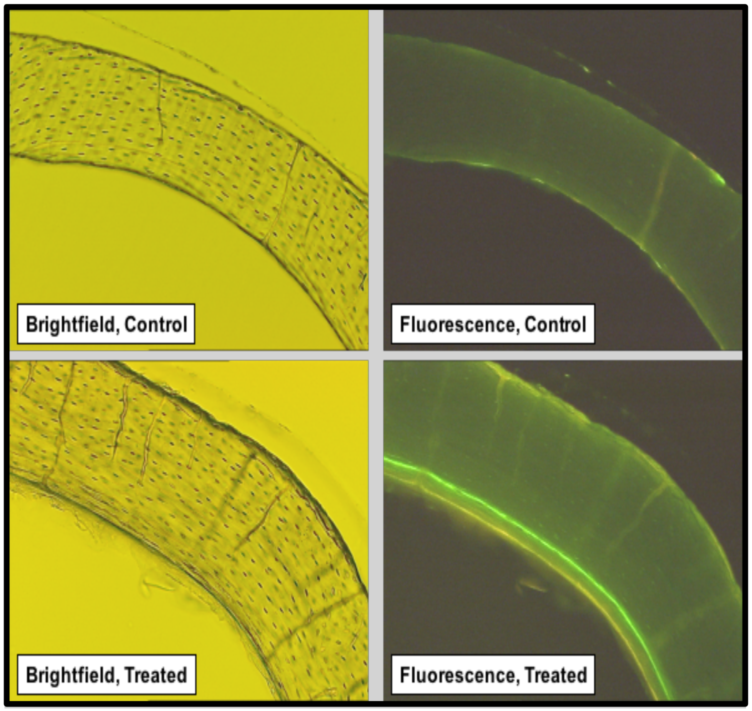

Brightfield (left) and epifluorescence (right) images showing minimal fluorochrome incorporation adjacent to the marrow cavity in control bones (left) but robust endocortical bone formation with NOTUM inhibition (right). The mice were injected with calcein (green) and demeclocycline (yellow) fluorochromes on days 7 and 21, respectively, during 28 days of treatment. Numerical values for this study are provided in Figure 7.

Supplementary Figure 14: Treatment with NOTUM antibody 2.78.33 increased serum levels of the biochemical bone formation marker type 1 procollagen N-terminal propeptide (P1NP)

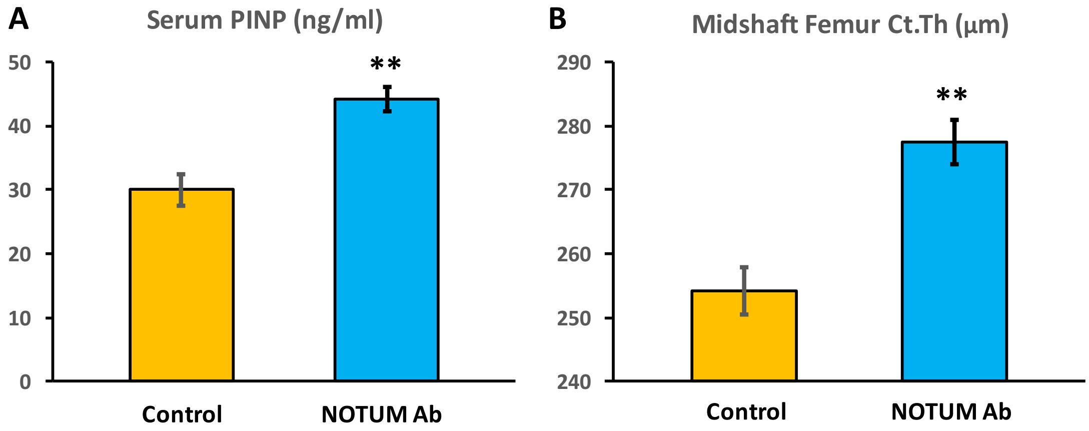

Male mice (32 weeks of age) were treated with Ab 2.78.33 (10 mg/kg) given weekly. **A, B** Serum levels of P1NP were analyzed after 7 days while femur cortical bone thickness was measured 28 days after study start. All values are means ± SEM.

** $P < 0.01$ compared to control

Supplementary Figure 15: Illustration of the separation of trabecular and cortical bone in the vertebral body using microCT.

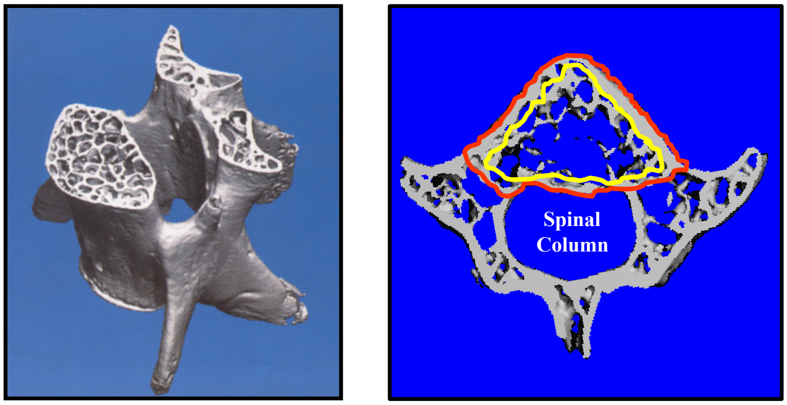

Fifth lumbar vertebrae were scanned at 8 μm voxel size using a Scanco μCT40 scanner. In addition to standard contouring of trabecular bone (shown in yellow; right panel) a second contour of the external surface of the vertebral body (shown in red) was performed. Total bone volume/total volume (BV/TV) is defined as bone inside the red contour. Trabecular BV/TV is defined as bone inside the yellow contour. Cortical BV/TV is defined as the difference between total BV/TV and trabecular BV/TV. BV/TV values for cortical and trabecular bone in wild-type mice are similar, as was observed in human vertebral bodies measured by dissection (Nottesad).

Nottestad SY, Baumel JJ, Kimmel DB, Recker RR, Heaney RP. The proportion of trabecular bone in human vertebrae. J Bone Miner Res. 1987; 2:221-229.

<u>Supplementary Figure 16</u>: MicroCT Scans of Mouse Femoral Neck

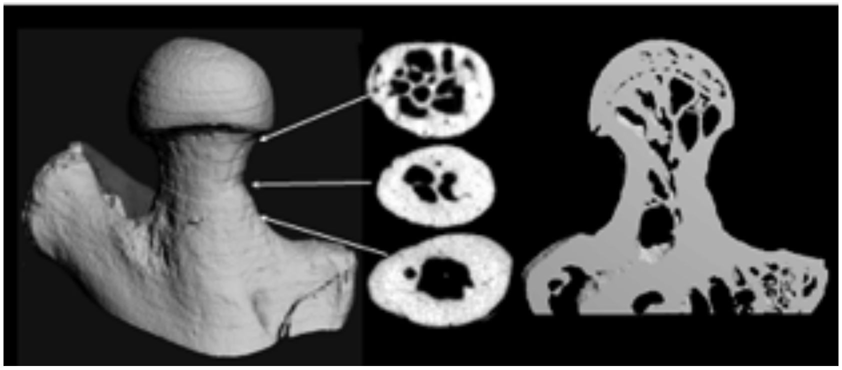

Dissected proximal femurs were analyzed by microCT using a Scanco Medical µCT40 (Brüttisellen, Switzerland). Scan parameters included an X-ray tube voltage of 55 keV, a current of 145 microamperes, an integration time of 200 milliseconds, and an analysis threshold of 240, with 6-micrometer isotropic voxel size. Careful positioning of the bones in the sample holder to obtain vertical orientation of the neck is critical. The entire neck (≈150 slices) is scanned and a standard 2-D analysis is generated. From the 2-D analysis, the slice having the lowest total area is identified. A standard 3-D analysis is then performed using a total of 20 slices with the middle slice having the lowest total area in the 2-D analysis. To avoid subjective bias, no attempt is made to separate trabecular from cortical bone. Using the Direct, model-independent mode, total volume (TV), bone volume (BV) and BV/TV are determined. Total and bone areas are defined from the corresponding volumes divided by the 120-micrometer length analyzed.

In separate studies, femoral neck BV/TV is elevated in *Sost*$^{-/-}$ mice and following teriparatide treatment, reduced in *Lrp5*$^{-/-}$ mice, following ovariectomy and with aging.

Brommage, R., Jeter-Jones, S., Xiong, W., Champ, R. & Liu, J. Mouse femoral neck architecture determined by microCT reflects skeletal architecture observed at other bone sites. *Journal of Bone and Mineral Research* 28 (Suppl 1), S347-S348 (2013).

<u>Supplementary Table 1</u>: Midshaft Femur Cortical Bone Thickness in Male *Notum* KO Mouse Cohorts

Cohort (Birth)	Age (weeks)	WT Mice N	WT Mice Ct.Th (µm)	KO Mice N	KO Mice Ct.Th (µm)	KO/WT Increase
June 2006	3.7	9	143 ± 6	5	157 ± 4	9%
June 2009	4	14	159 ± 4	13	188 ± 5	18%
August 2004	16	10	257 ± 7	10	329 ± 6	28%
Throughout 2005	16	23	267 ± 5	17	312 ± 6	17%
January 2008	16	9	301 ± 6	9	339 ± 13	13%
June 2009	16	11	246 ± 7	10	270 ± 7	10%
August 2011	18	10	227 ± 6	10	266 ± 7	17%
April 2004	19	7	263 ± 7	5	318 ± 6	21%
December 2004	26	10	272 ± 5	9	322 ± 11	17%
January 2007	33	6	249 ± 3	6	323 ± 14	30%
June 2009	34	11	248 ± 4	6	282 ± 7	14%
July 2011	34	9	249 ± 6	9	303 ± 5	22%
October 2011	35	21	264 ± 3	21	301 ± 6	14%
January 2005	52	6	273 ± 10	8	330 ± 14	21%
All Adult Males	≥ 16	133	260 ± 25 (SD)	120	307 ± 34 (SD)	18%

Midshaft Femur Cortical Bone Thickness in Female *Notum* KO Mouse Cohorts

Cohort (Birth)	Age (weeks)	WT Mice N	WT Mice Ct.Th (µm)	KO Mice N	KO Mice Ct.Th (µm)	KO/WT Increase
June 2006	3.4	5	131 ± 3	5	142 ± 5	9%
June 2009	4	9	159 ± 2	10	179 ± 5	12%
December 2004	8	18	216 ± 5	11	231 ± 7	7%
May 2011	14	6	232 ± 5	6	273 ± 4	18%
Throughout 2005	16	22	240 ± 4	18	298 ± 5	24%
January 2008	16	9	255 ± 6	9	327 ± 7	28%
June 2009	16	10	232 ± 3	10	263 ± 7	13%
April 2004	17	18	242 ± 4	7	295 ± 10	22%
July 2011	18	14	252 ± 6	12	285 ± 7	13%
June 2006	26	13	249 ± 8	14	324 ± 6	30%
January 2007	42	13	284 ± 8	7	333 ± 11	17%
July 2011	43	13	254 ± 8	9	307 ± 11	21%
All Adult Females	≥ 14	118	249 ± 25 (SD)	92	301 ± 32 (SD)	21%

All data are means ± SEMs for the number of bones indicated, except for adult totals with standard deviations (SD) presented.

<u>Supplementary Table 2</u>: Cortical Bone Thickness Phenotypes in *Notum* KO Mouse Cohorts

Cohort (Birth)	Bone	Sex	Age (weeks)	WT Mice N	WT Mice Ct.Th (µm)	KO Mice N	KO Mice Ct.Th (µm)	KO/WT Increase	P Value
August 2004	Midshaft Humerus	Male	16	10	221 ± 5	10	247 ± 2	19%	P < 0.001
August 2004	Midshaft Tibia	Male	16	10	278 ± 8	10	326 ± 5	17%	P < 0.001
August 2004	Tibia at Fibula	Male	16	10	305 ± 6	10	338 ± 6	11%	P = 0.001
September 2004	Midshaft Humerus	Female	8	11	187 ± 2	11	204 ± 4	9%	P < 0.001
August 2011	Midshaft Humerus	Male	18	10	229 ± 5	10	255± 3	11%	P < 0.001
August 2011	Midshaft Tibia	Male	18	10	244 ± 3	10	269± 3	10%	P < 0.001
August 2011	Midshaft Radius	Male	18	10	200 ± 3	10	235± 6	12%	P < 0.001
October 2011	Pelvis	Male	35	21	221 ± 3	21	247 ± 5	12%	P < 0.001

All data are means ± SEMs for the number of bones indicated. Pelvic bones were analyzed one quarter of the distance from the acetabulum to top of the iliac crest (see photo below).

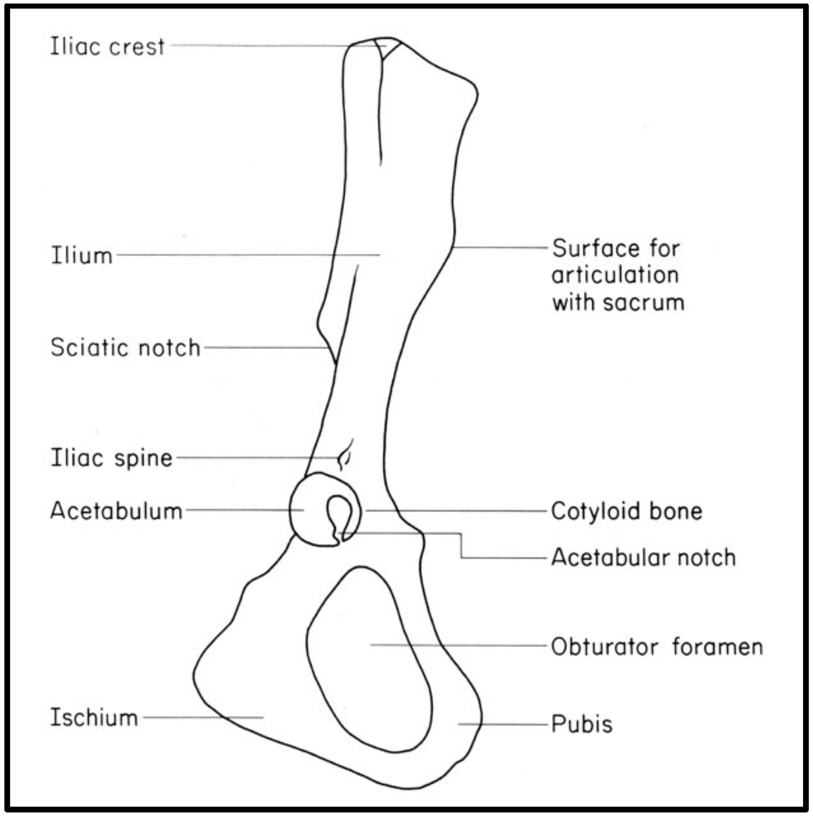

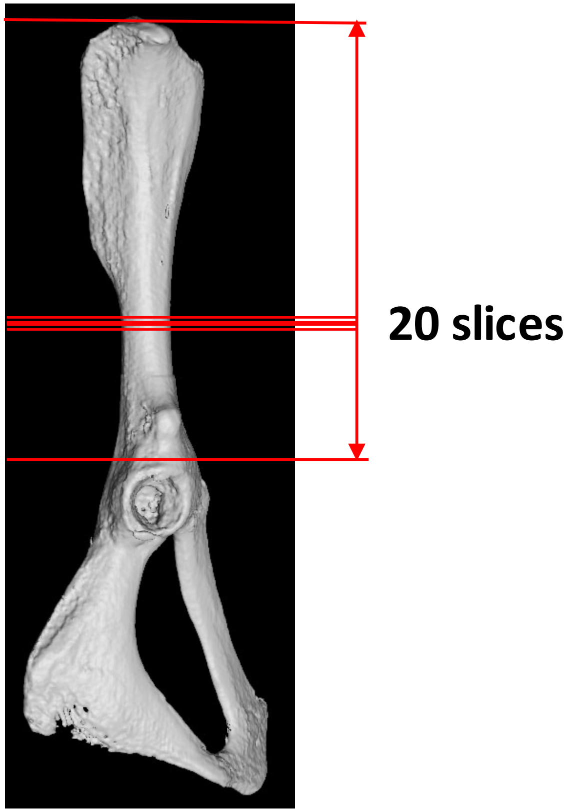

http://www.informatics.jax.org/cookbook/figures/figure37.shtml

Supplementary Table 3: Femoral Neck and Femur Length Data in *Notum*$^{-/-}$ Mice

Bone Measurement	Sex	Age	N	Genotype	Value
Femoral Neck BV/TV (%)	Male	17 weeks	9	Wild-type	85.9 ± 0.6
Femoral Neck BV/TV (%)	Male	17 weeks	10	*Notum*$^{-/-}$	89.7 ± 0.8**
Femur Length (mm)	Male	16 weeks	23	Wild-type	16.5 ± 0.1
Femur Length (mm)	Male	16 weeks	17	*Notum*$^{-/-}$	16.4 ± 0.1
Femur Length (mm)	Female	16 weeks	22	Wild-type	16.0 ± 0.1
Femur Length (mm)	Female	16 weeks	18	*Notum*$^{-/-}$	15.8 ± 0.1
Femur Length (mm)	Male	26 weeks	11	Wild-type	15.8 ± 0.1
Femur Length (mm)	Male	26 weeks	10	*Notum*$^{-/-}$	15.9 ± 0.2
Femur Length (mm)	Female	26 weeks	10	Wild-type	15.4 ± 0.1
Femur Length (mm)	Female	26 weeks	10	*Notum*$^{-/-}$	15.4 ± 0.1

Values are means ± SEM. ** $p < 0.01$ vs WT.

<u>Supplementary Table 4</u>: Soft Tissue Histopathology in *Notum*$^{-/-}$ Mice

No histopathological abnormalities were observed in the following 40 soft tissues examined in 8 *Notum*$^{-/-}$ compared with 4 wild-type littermate mice at 40 weeks of age.

Heart, skeletal muscle, aorta, lung, mediastinal lymph node, kidney, renal lymph node, trachea, thyroid, parathyroid gland, adrenal gland, pituitary gland, thymus, salivary gland, cervical lymph node, esophagus, stomach, pancreas, duodenum, jejunum, ileum, cecum, colon, rectum, mesenteric lymph node, liver, gallbladder, spleen, brain, spinal cord, eyes, Harderian gland, urinary bladder, uterus, ovaries, fallopian tube, prostate, testes, epididymis, seminal vesicle, vas deferens, urethral glands, skin, mammary gland, mammary lymph node, adipose tissue, blood, bone marrow, ear

<u>Supplementary Table 5</u>: Clinical Chemistry and Blood Cell Count Evaluations in Adult *Notum* KO Mice

Clinical Chemistry Parameters

Parameter	Units	Sex	WT	KO	P Value	Δ KO
N		Male	22	19	-	-
N		Female	20	20	-	-
Total Protein	g/dl	Male	5.58 ± 0.07	6.03 ± 0.07	P < 0.001	↑ 8%
Total Protein	g/dl	Female	5.51 ± 0.07	6.04 ± 0.10	P < 0.001	↑ 10%
Albumin	g/dl	Male	3.71 ± 0.06	3.44 ± 0.07	P = 0.004	↓ 7%
Albumin	g/dl	Female	3.88 ± 0.04	3.51 ± 0.07	P < 0.001	↓ 10%
Globulins	g/dl	Male	1.86 ± 0.06	2.60 ± 0.08	P < 0.001	↑ 40%
Globulins	g/dl	Female	1.63 ± 0.08	2.55 ± 0.11	P < 0.001	↑ 56%
Urea Nitrogen	mg/dl	Male	25.5 ± 1.2	28.3 ± 1.1	P = 0.11	
Urea Nitrogen	mg/dl	Female	25.0 ± 1.1	24.6 ± 1.1	P = 0.77	
Creatinine	mg/dl	Male	0.13 ± 0.01	0.15 ± 0.01	P = 0.06	
Creatinine	mg/dl	Female	0.13 ± 0.01	0.14 ± 0.01	P = 0.77	
Total Bilirubin	mg/dl	Male	0.37 ± 0.07	0.32 ± 0.04	P = 0.55	
Total Bilirubin	mg/dl	Female	0.48 ± 0.06	0.34 ± 0.06	P = 0.10	
Uric Acid	mg/dl	Male	1.70 ± 0.21	1.95 ± 0.20	P = 0.40	
Uric Acid	mg/dl	Female	1.96 ± 0.23	1.62 ± 0.21	P = 0.28	
AST	U/l	Male	161 ± 25	170 ± 39	P = 0.85	
AST	U/l	Female	128 ± 15	121 ± 12	P = 0.74	

Non-fasted serum was obtained at 16 weeks of age. AST = aspartate transaminase. Statistical evaluations by Student's t-test. All data are means ± SEMs.

Blood Cell Count Parameters

Parameter (units)	WT	KO	P Value	Δ KO
WBCs (10^3/µl)	7.94 ± 1.04	11.80 ± 1.06	P = 0.02	↑ 49%
Neutrophils (10^3/µl)	0.93 ± 0.21	1.84 ± 0.18	P = 0.003	↑ 97%
Lymphocytes (10^3/µl)	6.83 ± 0.97	9.45 ± 0.85	P = 0.05	↑ 38%
Monocytes (10^3/µl)	0.16 ± 0.04	0.50 ± 0.10	P = 0.004	↑ 3-fold
Eosinophils (10^3/µl)	0.009 ± 0.003	0.008 ± 0.003	P = 0.83	
Basophils (10^3/µl)	0.008 ± 0.002	0.011 ± 0.004	P = 0.46	
RBCs (10^6/µl)	10.8 ± 0.1	10.0 ± 0.1	P < 0.001	↓ 8%
Hemoglobin (g/dl)	15.8 ± 0.2	14.6 ± 0.2	P < 0.001	↓ 7%
Hematocrit (%)	44.2 ± 0.5	41.1 ± 0.5	P < 0.001	↓ 7%

Blood was obtained at 26 weeks of age from female mice with N = 10 for each genotype. Statistical evaluations by Student's t-test All data are means ± SEMs.

<u>Supplementary Table 6</u>: Static Histomorphometry of LV4 Trabecular Bone with OVX and LP-9220565 Treatment

Parameter	Sham Control	Sham Plus LP-9220556	OVX Control	OVX Plus LP-922056	OVX Effect	Treatment Effect	Interaction P Value
Tb BV/TV (%)	48.4 ± 2.3	48.9 ± 1.7	20.3 ± 1.9	24.8 ± 2.5	P < 0.001	P = 0.26	P = 0.34
Tb Thickness (μm)	64.7 ± 3.3	60.2 ± 2.4	39.7 ± 1.4	45.7 ± 2.5	P < 0.001	P = 0.66	P = 0.06
Tb Number (1/N)	9.04 ± 0.31	9.77 ± 0.19	6.02 ± 0.36	6.48 ± 0.47	P < 0.001	P = 0.10	P = 0.70
Tb Oc.S/BS (%)	0.38 ± 0.7	0.28 ± 0.06	0.65 ± 0.12	1.01 ± 0.26	P = 0.002	P = 0.38	P = 0.14
Tb Ob.S/BS (%)	7.71 ± 1.29	8.55 ± 1.29	6.72 ± 0.87	10.43 ± 1.53	P = 0.22	P = 0.23	P = 0.94
Tb OS/BS (%)	7.31 ± 1.30	8.10 ± 1.57	6.31 ± 0.82	9.62 ± 1.44	P = 0.89	P = 0.12	P = 0.38

Rats underwent ovariectomy or sham surgery at 21 weeks of age LP-922056 or vehicle were given by daily oral gavage from 58 through 76 weeks of age. LV4 trabecular bone was examined by standard histomorphometric techniques. Data are means ± SEM for 12 bones per group with statistical analysis by two-factor ANOVA. Histomorphometric analyses were performed in the laboratory of Thomas J. Wronski at the University of Florida.

<u>Supplementary Table 7</u>: Published Teriparatide Benchmark Studies

First Author	Treatment Group	N	Femur Ct.Th (µm)	TPTD Effect (Percent)
Iwaniec	Male – WT – Vehicle	11	252 ± 2	
	Male – WT – TPTD	12	291 ± 7	15%
Iwaniec	Male – KO – Vehicle	13	228 ± 4	
	Male – KO – TPTD	14	255 ± 7	12%
Iwaniec	Female – WT – Vehicle	13	250 ± 5	
	Female – WT – TPTD	12	287± 8	15%
Iwaniec	Female – KO – Vehicle	13	214 ± 5	
	Female – KO – TPTD	13	245 ± 5	14%
Cui	Sham-Surgery – Vehicle	12	223 ± 3	
Cui	Sham-Surgery – TPTD	9	256 ± 4	15%
Cui	OVX – Vehicle	12	221 ± 5	
Cui	OVX – TPTD	8	258 ± 6	17%

Both studies were performed at Lexicon examining teriparatide (TPTD) at a daily subcutaneous dose of 80 µg/kg using identical procedures employed for the NOTUM studies. For the Iwaniec study, male and female *Lrp5* KO mice and their wild-type littermates (C57BL/6J – 129SvEv F2 hybrids) were treated with TPTD or vehicle every second day for 6 weeks, starting at 20 to 22 weeks of age. For the Cui study, ovariectomized and sham-surgery control mice (C57BL/6J, with surgery at 16 weeks of age) were treated with TPTD or vehicle daily for 6 weeks, starting at 67 weeks of age. Midshaft femur cortical thickness (Ct.Th) values (measured by microCT) are means ± SEM for the number of bones indicated.

Iwaniec UT, Wronski TJ, Liu J, Rivera MF, Arzaga RR, Hansen G, Brommage R. PTH stimulates bone formation in mice deficient in Lrp5. *J Bone Miner Res.* **22**, 394-402 (2007).

Cui Y, Niziolek PJ, MacDonald BT, Zylstra CR, Alenina N, Robinson DR, Zhong Z, Matthes S, Jacobsen CM, Conlon RA, Brommage R, Liu Q, Mseeh F, Powell DR, Yang QM, Zambrowicz B, Gerrits H, Gossen JA, He X, Bader M, Williams BO, Warman ML, Robling AG. Lrp5 functions in bone to regulate bone mass. *Nat Med.* **17**, 684-691 (2011).

Supplementary Table 8: Pharmacokinetic Data Providing Drug Exposure Values

Figure	Animal	Compound	Dose	Administration	Day	Time	[Serum]
2A – 2E	WT Mouse	LP-922056	10 mg/kg	Diet	7	9:00	52 µM
2F	WT Mouse	LP-922056	30 mg/kg	Diet	10	8:00	423 µM
2F	HUM Mouse	LP-922056	30 mg/kg	Diet	10	8:00	425 µM
2F	WT Mouse	LP-922056	30 mg/kg	Diet	10	16:00	313 µM
2F	HUM Mouse	LP-922056	30 mg/kg	Diet	10	16:00	307 µM
2G, 3K-3N	WT Mouse	LP-914822	5 mg/kg	Oral Gavage BID	7	Plus 10 Hours	26 µM
2G, 3K-3N	WT Mouse	LP-914822	5 mg/kg	Oral Gavage BID	8	Plus 13 Hours	38 µM
2G, 3K-3N	WT Mouse	LP-914822	15 mg/kg	Oral Gavage BID	7	Plus 10 Hours	96 µM
2G, 3K-3N	WT Mouse	LP-914822	15 mg/kg	Oral Gavage BID	8	Plus 13 Hours	124 µM
2G, 3K-3N	WT Mouse	LP-914822	30 mg/kg	Oral Gavage BID	7	Plus 10 Hours	140 µM
2G, 3K-3N	WT Mouse	LP-914822	30 mg/kg	Oral Gavage BID	8	Plus 13 Hours	189 µM
2H	WT Mouse	LP-935001	3 mg/kg	Oral Gavage	7	Plus 2 Hours	8 µM
2H	WT Mouse	LP-935001	10 mg/kg	Oral Gavage	7	Plus 2 Hours	63 µM
2H	WT Mouse	LP-935001	30 mg/kg	Oral Gavage	7	Plus 2 Hours	198 µM
S2C	WT Mouse	LP-922056	1 mg/kg	Diet	7	8:00	7 µM
S2C	WT Mouse	LP-922056	3 mg/kg	Diet	7	8:00	20 µM
S2C	WT Mouse	LP-922056	9 mg/kg	Diet	7	8:00	72 µM
S2D	WT Mouse	LP-922056	3 mg/kg	Oral Gavage 2X/Wk	14	Plus 6 hours	71 µM
S2D	WT Mouse	LP-922056	10 mg/kg	Oral Gavage 2X/Wk	14	Plus 6 hours	208 µM
S2D	WT Mouse	LP-922056	30 mg/kg	Oral Gavage 2X/Wk	14	Plus 6 hours	399 µM
3A-3F	WT Mouse	LP-922056	30 mg/kg	Oral Gavage		No Measurements	
5A-5K	Control Rat	LP-922056	30 mg/kg	Oral Gavage	26	Plus 6 hours	599 µM
5A-5K	OVX Rat	LP-922056	30 mg/kg	Oral Gavage	26	Plus 6 hours	587 µM
5A-5K	Control Rat	LP-922056	30 mg/kg	Oral Gavage	55	Plus 6 hours	747 µM
5A-5K	OVX Rat	LP-922056	30 mg/kg	Oral Gavage	55	Plus 6 hours	858 µM
5A-5K	Control Rat	LP-922056	30 mg/kg	Oral Gavage	111	Plus 6 hours	504 µM
5A-5K	OVX Rat	LP-922056	30 mg/kg	Oral Gavage	111	Plus 6 hours	500 µM

Serum compound levels were measured by LCMS using standard techniques on the Study Days and Times indicated. For Figure 2G, 3K-3N, mice were routinely dosed daily at 7:00 and 18:00 and for PK data were bled on Day 7 at 17:00 and on Day 8 at 7:00 prior to routine dosing. For Figure S2D, mice were dosed on Mondays and Thursdays only.

Supplementary Table 9: Reproducibility of LP-914822 Mouse Pharmacology Studies

Eighteen pharmacology studies examining male C57BL/6J – 129SvEv F1 hybrid mice treated with LP-914822 given via diet at a target dose of 30 mg/kg were performed. Five of these studies examined hypotheses related to the actions of LP-914822 and 13 studies included LP-914822 as a positive control group when testing additional NOTUM inhibitors.

Study Date	Age (weeks)	BW (grams)	Dose (mg/kg)	Duration (days)	Control N	LP-914822 N	Treatment Effect	Study Groups
February 2009	12	31.3	28	35	12	9	12.4%	N = 5
October 2009	9	28.0	26	28	12	12	11.0%	N = 4
February 2010	11	32.1	28	28	12	12	11.6%	N = 8
January 2011	10	30.8	27	28	16	12	14.1%	N = 5
February 2011	10	31.0	23	28	16	12	5.2%	N = 5
March 2011	11	31.6	27	28	12	12	8.2%	N = 4
April 2011	9	29.4	29	28	16	12	12.0%	N = 5
May 2011 #1	11	33.3	31	28	16	12	14.6%	N = 5
May 2011 #2	12	32.6	27	28	16	12	9.8%	N = 5
June 2011	12	32.7	28	25	16	12	12.7%	N = 5
July 2011 #1	13	37.5	29	28	12	12	13.3%	N = 4
July 2011 #2	12	36.3	29	24	16	12	10.8%	N = 5
July 2011 #3	12	35.9	29	28	16	12	6.0%	N = 5
August 2011 #1	13	37.1	31	25	16	12	10.4%	N = 5
August 2011 #2	12	36.0	33	28	16	12	11.2%	N = 5
September 2011	12	34.8	33	28	14	14	9.9%	N = 4
December 2011	13	35.3	27	24	16	12	7.5%	N = 5
January 2012	13	34.1	30	25	16	12	12.3%	N = 5
Means, N = 18	12	33	29	27	N = 268	N = 215	10.7%	N = 4 to 8

For each study, start ages and body weighs (BW), study duration and numbers of both control and LP-914822-treated mice are provided. Study groups refer to the total number of mouse groups examined, with data in this table obtained from the negative and positive control groups. Mice were treated by mixing LP-914822 into purified low fat diet. Doses were calculated knowing mouse BWs, the diet concentration of LP-914822, and food consumption measured several times weekly.

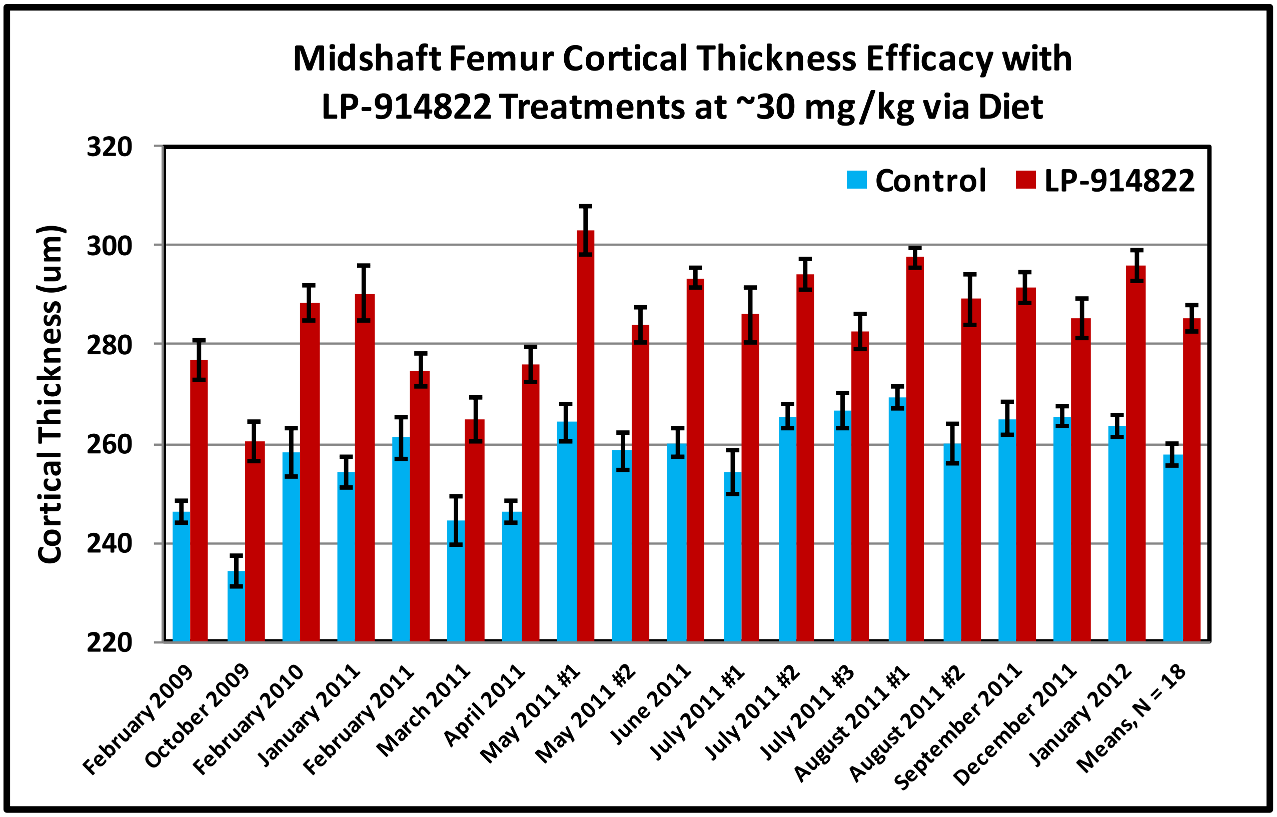

Data are means ± SEMs for the numbers of bones listed in the table.
